# A specific type of insulin-like peptide regulates the conditional growth of a beetle weapon

**DOI:** 10.1371/journal.pbio.3000541

**Published:** 2019-11-27

**Authors:** Yasukazu Okada, Masako Katsuki, Naoki Okamoto, Haruna Fujioka, Kensuke Okada

**Affiliations:** 1 Department of Biological Sciences, Tokyo Metropolitan University, Hachioji, Tokyo, Japan; 2 Laboratory of Applied Entomology, Graduate School of Agricultural and Life Sciences, The University of Tokyo, Tokyo, Japan; 3 Department of Entomology, Institute for Integrative Genome Biology, University of California, Riverside, Riverside, California, United States of America; 4 Department of General Systems studies, Graduate School of Arts and Sciences, The University of Tokyo, Komaba, Tokyo, Japan; 5 Graduate School of Environmental and Life Science, Okayama University, Okayama, Japan; University of Lausanne, SWITZERLAND

## Abstract

Evolutionarily conserved insulin/insulin-like growth factor (IGF) signaling (IIS) has been identified as a major physiological mechanism underlying the nutrient-dependent regulation of sexually selected weapon growth in animals. However, the molecular mechanisms that couple nutritional state with weapon growth remain largely unknown. Here, we show that one specific subtype of insulin-like peptide (ILP) responds to nutrient status and thereby regulates weapon size in the broad-horned flour beetle *Gnatocerus cornutus*. By using transcriptome information, we identified five *G*. *cornutus* ILP (*GcorILP1–5*) and two *G*. *cornutus* insulin-like receptor (*GcorInR1*, *-2*) genes in the *G*. *cornutus* genome. RNA interference (RNAi)-mediated gene silencing revealed that a certain subtype of ILP, *GcorILP2*, specifically regulated weapon size. Importantly, *GcorILP2* was highly and specifically expressed in the fat body in a condition-dependent manner. We further found that *GcorInR1* and *GcorInR2* are functionally redundant but that the latter is partially specialized for regulating weapon growth. These results strongly suggest that *GcorILP2* is an important component of the developmental mechanism that couples nutritional state to weapon growth in *G*. *cornutus*. We propose that the duplication and subsequent diversification of IIS genes played a pivotal role in the evolution of the complex growth regulation of secondary sexual traits.

## Abstract 2

Animals can evolve sexually selected weapons and ornaments as the exaggerations of specific body parts, often linked to nutritional status. This study shows that in the broad-horned flour beetle, one insulin-like peptide (among the five encoded by their genome) was found to specifically regulate weapon growth. Functional diversification of ligand peptides may account for the ecological divergence of sexually selected exaggerated traits.

## Introduction

Sexual selection often leads animals to evolve strikingly exaggerated male ornaments and weapons [1], which are often characterized by heightened nutritional sensitivity during growth. Intraspecific variation of secondary sexual trait size is in turn often associated with condition-dependent mating tactics, such as fighting, sneaking, or dispersal [2–4]. Therefore, these traits are most exaggerated in the largest and best-conditioned individuals but rudimentary in low-conditioned males, commonly resulting in positive allometries of weapons and ornaments [5,6]. Such conditional expression of sexual traits is a good example of the adaptive evolution of phenotypic complexity [7]. Consequently, conditional growth is a fundamental mechanism underlying the evolution of exaggerated phenotypes and their complex development; however, an in-depth understanding of these mechanisms is lacking [8,9].

The major nutrient-dependent endocrine pathway, insulin/insulin-like growth factor (IGF) signaling (IIS), has been identified as a mechanism that can link exaggerated trait growth and nutritional condition [9–11]. In the Japanese rhinoceros beetle, *Trypoxylus dichotomus*, down-regulation of IIS by the knockdown (KD) of receptor gene (*InR*) caused a dramatic reduction in horn length in adults but resulted in only a slight reduction in wing and genital size [10]. In a dung beetle, *Onthophagus taurus*, KD of transcription factor *FOXO* but not *InR* affected horn length [12]. This ancient and conserved pathway that couples growth with available nutrients is considered to have been repeatedly co-opted in linages that experienced strong sexual selection [10]. However, the deployed genes may be more diverse than previously expected.

The most upstream central players in IIS are insulin-like peptides (ILPs), which include insulin and IGFs in mammals [13] and multiple ILPs in insects [14,15]. In both mammals and insects, the production, secretion, and action of insulin/IGFs/ILPs are mainly regulated by the nutritional status [16–18]. They activate receptor tyrosine kinases (i.e., insulin receptor and IGF type-I receptor) in mammals and InRs in insects to accelerate cellular growth, proliferation, and metabolism [14,15,19,20].

Although this signal transduction mechanism is conserved across taxa, individual gene members of this pathway, especially ligands and receptors, may have undergone substantial diversification. For example, several insect species possess two or three InRs [14,21]. Interestingly, in the brown planthopper, two receptors alternately regulate the polyphenic development of short- and long-winged morphs [22]. Further, in the yellow fever mosquito and clonal raider ant, a certain type of ILP is known to regulate reproductive status [23–26]. Recent genome-wide investigations implied that insect ILPs are also highly diversified within and across species. Relatively basal clades, such as Orthoptera, possess only one ILP [27], whereas there are up to four ILPs in the red flour beetle [28], eight in the fruit fly and yellow fever mosquito [29,30], 10 in the pea aphid [31], and more than 40 in the silkworm [32]. In insects, ILP production and secretion from the brain are considered as the principal mechanisms for nutrient-dependent systemic growth and metabolism [14,15,18]. However, other non-neurosecretory ILPs also have important growth functions in specific stages and/or tissues [15,33–35].

Given that gene-duplications play pivotal roles in the evolution of phenotypic complexity [36,37], the frequent multiplications of insect ILPs and InRs suggest the potential for functional diversification, such as trait- and stage-specific functions. So far, however, whether and how different types of ILPs and InRs functionally contribute to phenotypic complexity remain largely unknown. Here, we focused on the developmental plasticity of a beetle weapon and tested the hypothesis that specific types of IIS ligands and receptors regulate weapon growth.

**Fig 1:**
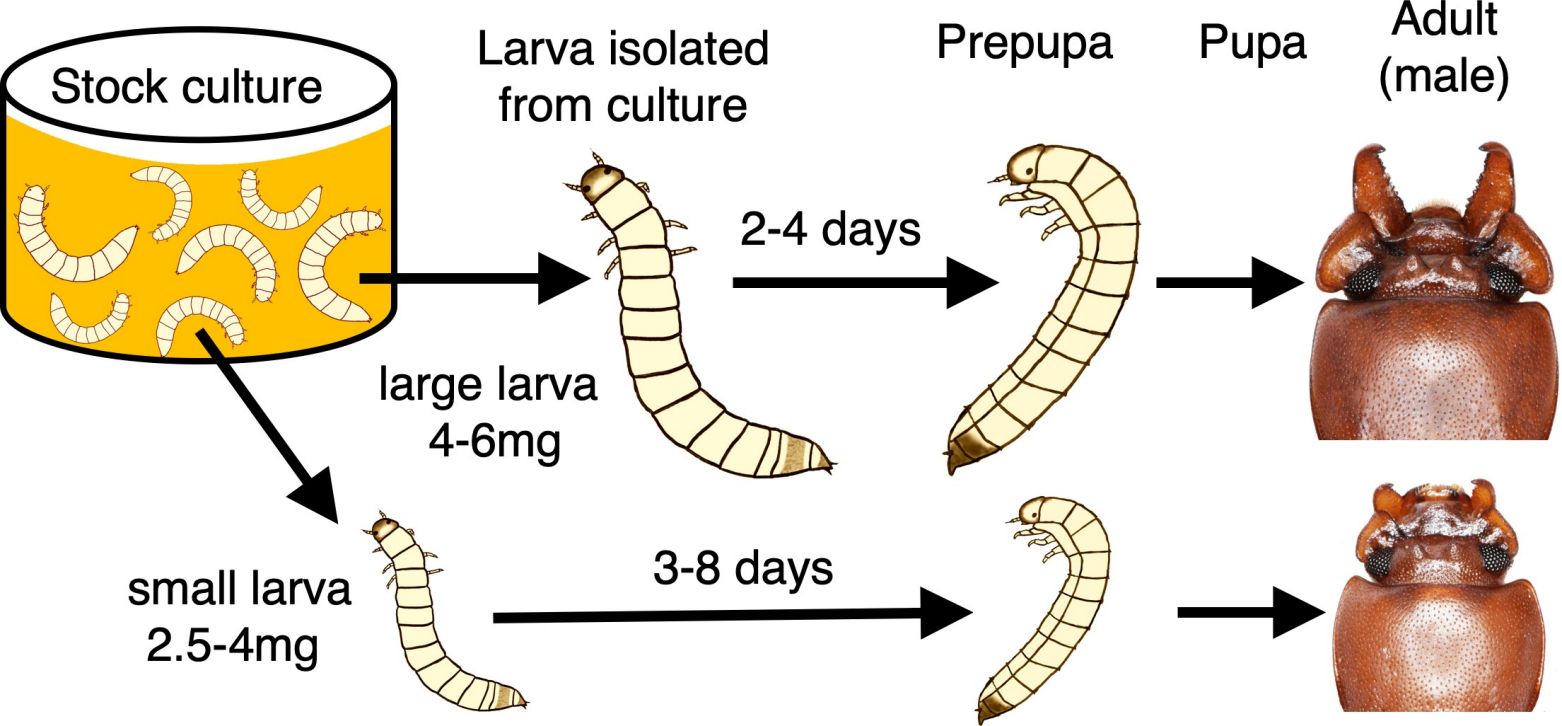
Development of *G*. *cornutus* and experimental procedure.

Males of the broad-horned flour beetle *G*. *cornutus* have exaggerated mandibles used in male–male combat (Fig 1), and males possessing larger mandibles have an advantage in these contests [38]. Large males have disproportionately larger mandibles (i.e., positive allometry), and this variation is mostly generated by the amount and quality of larval diet [38,39]. Therefore, the male mandibles of *G*. *cornutus* are a good model for the heightened conditional growth of a secondary sexual trait. Here, using *G*. *cornutus*, we investigated the molecular basis of this conditional growth by identifying the specific peptides that link nutritional condition with weapon growth.

Broad-horned flour beetle larvae were cultured in groups (approximately *n* = 200) for 30–60 days in whole-wheat flour medium. This high-density culture condition inhibits the pupation of larvae, but the isolation from the culture immediately induces pupation. The beginning of pupation is visually detectable by the characteristic L-shape of prepupa. For prepupation, large individuals (>4 mg) take 2‒4 days, whereas small individuals (2.5‒4 mg) take 3‒8 days. See details in S1 Fig and Materials and Methods.

## Results

### The schedule of metamorphic development in *G*. *cornutus*

Prior to molecular developmental analyses, we determined the detailed patterns of development and its condition dependence in *G*. *cornutus*. Unsurprisingly, larval size variation originated from the amount of dietary intake before metamorphosis, and the nutritional condition eventually determines the male sexual phenotype in this species (Fig 1). In *G*. *cornutus*, the timing of pupation is determined by a “critical size” and food availability, similar to many other holometabolous insects [40]. From the preliminary experiment, larvae (30‒60 days from egg oviposition) weighing 2.5‒4 mg were defined as poorly fed “small individuals,” and those weighing >4 mg were defined as sufficiently fed “large individuals” (Fig 1, S1 Fig, also see Materials and Methods). Note that smaller larvae take longer for prepupation (Fig 1, S1 Fig). This negative relationship between larval weight and the time required for prepupation implies that small, low-conditioned larvae do not pupate immediately. Consequently, the developmental process slightly differed between large and small individuals in the following gene expression analyses.

### Identification and classification of ILPs and InRs in *G*. *cornutus*

The genome of the red flour beetle *Tribolium castaneum* contains four *ILP* (*TcILP1–4*) and two *InR* (*TcInR1* and *TcInR2*) genes [28,41]. Using *TcILP* and *TcInR* sequences as queries, *G*. *cornutus ILP* and *InR* candidates were retrieved by local BLASTx against the larval transcriptome [42].

**Fig 2:**
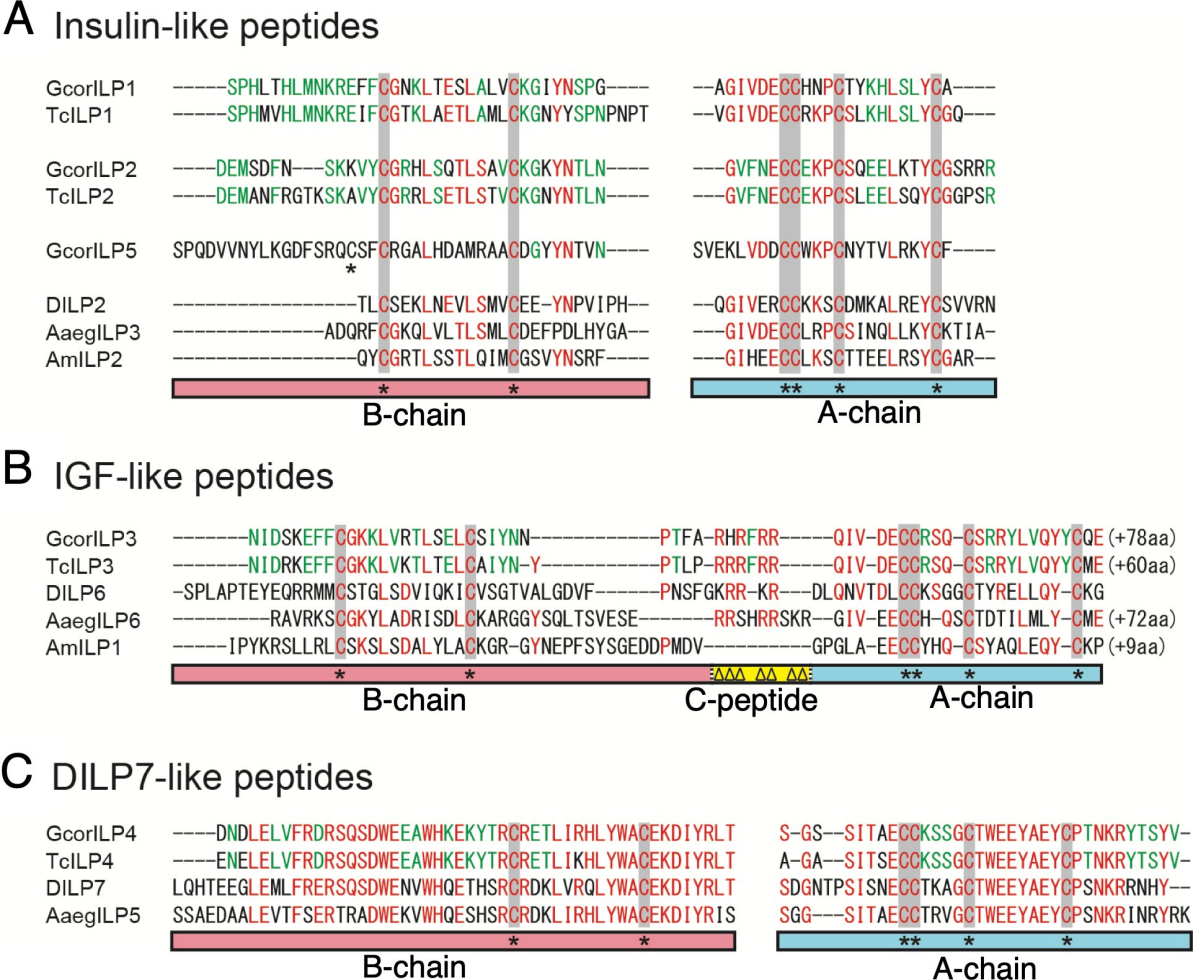
Predicted mature structures of insulin-like, IGF-like, and DILP7-like peptides in *G*. *cornutus*. Domain-based alignments of (A) insulin-like peptides, (B) IGF-like peptides, and (C) DILP7-like peptides from the broad-horned flour beetle (*G*. *cornutus*), red flour beetle (*T*. *castaneum*), fruit fly (*Drosophila melanogaster*), yellow fever mosquito (*Aedes aegypti*), and honey bee (*Apis mellifera*). Representative ILPs of the fruit fly (DILP2, -6, -7), mosquito (AaegILP3, -5, -6), and honey bee (AmILP1, -2) are shown. Highly conserved amino acid residues between all ILPs are shown in red, and those between orthologous ILPs in *G*. *cornutus* and *T*. *castaneum* are shown in green. Asterisks below the alignment denote conserved Cys residues. Note that signal peptide and C-peptide of insulin-like ILP are omitted to show the mature heterodimeric peptides. For the complete structure, see S6 Fig. AaegILP, *A*. *aegypti* ILP; AmILP, *A*. *mellifera* ILP; DILP, *Drosophila* ILP; GcorILP, *G*. *cornutus* ILP; IGF, insulin-like growth factor; ILP, insulin-like peptide; TcILP, *T*. *castaneum* ILP.

We identified five *ILP* genes in the *G*. *cornutus* transcript (Fig 2, S2 Fig, S1 Material). The amino acid phylogeny showed that four of the five *GcorILP* sequences had close similarity to the four *TcILPs*, indicating the clear orthologous relationships of these four genes (S3 Fig). We named these four ILP genes *GcorILP1*, *-2*, *-3*, and *-4* according to the nomenclature of *T*. *castaneum* [28]. The fifth ILP gene, which is novel in *G*. *cornutus*, was named *GcorILP5*. Although cross-species orthologs (ILP1–4) were clustered, only terminal nodes were supported by high bootstrap values; thus, evolutionary relationships among different orthologs were unclear (S3 Fig).

Since ILPs are highly diverged in their amino acid sequences except for some critical residues necessary for appropriate processing and tertiary structure, phylogeny-based analyses are often insufficient [34], and our data fit such a pattern. Therefore, based on domain structures and characteristic cysteine residues [18], we manually aligned and elucidated three ILP subtypes (Fig 2, S2 Fig) [18,28,43]: insulin-like peptides that contain long C-peptides with two dibasic cleavage sites at the boundary with the B- and A-chain (GcorILP1, GcorILP2, and GcorILP5; Fig 2A); IGF-like peptides that contain relatively short C-peptides, which usually lack a dibasic cleavage site at the boundary with the B- and/or A-domain (GcorILP3; Fig 2B); and *Drosophila* ILP (DILP) 7–like peptides that are highly conserved across insect linages (GcorILP4; Fig 2C). Note that mature structures after the removal of C-peptides are shown in Fig 2 for readability (see S2 Fig for full prepropeptide structure).

For receptors, we identified two *InRs* in *G*. *cornutus* transcripts. Construction of the protein phylogeny revealed that these two *GcorInR*s had clear orthologs in the *T*. *castaneum* genome (S4 Fig), and thus, they were named *GcorInR1* and *GcorInR2*.

### Condition-dependent expression of *GcorILP2* during development

**Fig 3:**
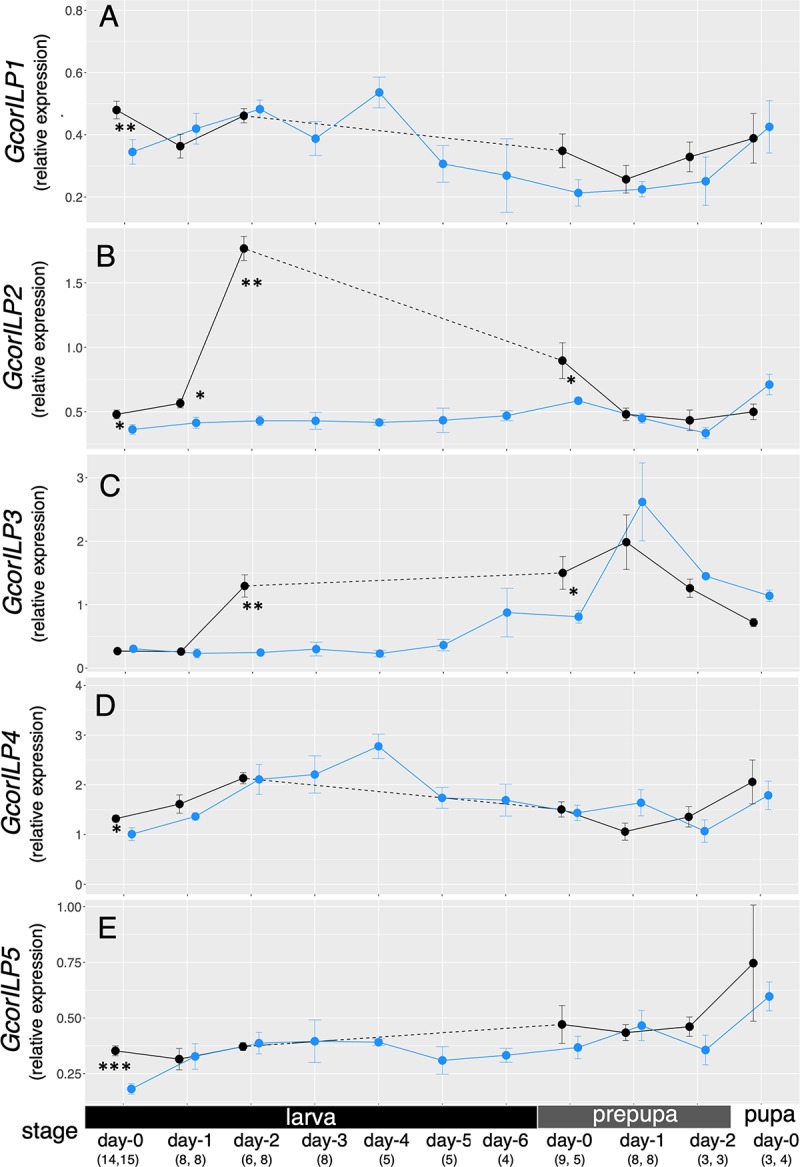
Developmental dynamics of insulin-like peptide transcription. *GcorILP* expression levels relative to *Gcorgapdh* were quantified by qPCR for the whole body. Black: large larvae; blue: small larvae. Results are presented as mean ± SE. Asterisks show significant differences between large and small larvae of the same stages (Mann-Whitney U test, *p < 0.05, **p < 0.01, ***p < 0.001). Larvae day-0 to day-6 corresponds to the days after larval isolation from culture. Prepupae day-0, -1, -2, and pupae day-0 correspond to the days after prepupation and pupation. Since large larvae take fewer days for prepupation (see Fig 1, S1 Fig), there were no large 3–6-day larvae (dashed line). The number of replicates is shown in parentheses. The underlying data in this figure are available from figshare (DOI: 10.6084/m9.figshare.9734780; https://figshare.com/s/609486022a3df39169bf). *Gcorgapdh*, *G*. *cornutus* glyceraldehyde 3-phosphate dehydrogenase; *GcorILP*, *G*. *cornutus* insulin-like peptide; qPCR, quantitative PCR.

To profile the developmental expression pattern of *GcorILPs*, we quantified the whole-body transcript levels from larval to pupal stages in the two different classes of conditioned samples: large and small individuals (preparation details in Fig 1 and Materials and Methods). The results revealed that *GcorILP2* had a clear peak in day-2 larvae (immediately before prepupation) only in the large individuals (Fig 3B). Notably, *GcorILP2* levels were constantly low in small larvae. Even if we consider that small larvae take longer for pupation (Fig 1, S1 Fig), there is no apparent peak during the course of metamorphic, postfeeding development in small larvae.

The increase in *GcorILP3* was also observed in day-2 larvae and the prepupal stage of large individuals; however, small larvae also exhibited a similar increase in *GcorILP3* around the prepupal stage (day-6 larvae and day-0 and day-1 prepupae; Fig 3C). These results indicate that IGF-like *GcorILP3* is maintained at low levels during larval stages but increases during metamorphosis, irrespective of larval size. This result is consistent with the dominant expression of IGF-like peptides during metamorphosis in *Bombyx* and *Drosophila* [33–35]. We did not find clear positive nutritional responses of other *ILP*s (*GcorILP1*, *GcorILP4*, and *GcorILP5*), although they were slightly heightened in day-0 larvae (Fig 3A, 3D and 3E). These results suggest that, among the five *GcorILPs*, the expression of *GcorILP2* was specifically positively correlated with nutritional condition during postfeeding metamorphic development.

### Sex and tissue specificities of *GcorILP2*: Condition-dependent expression in fat body

**Fig 4:**
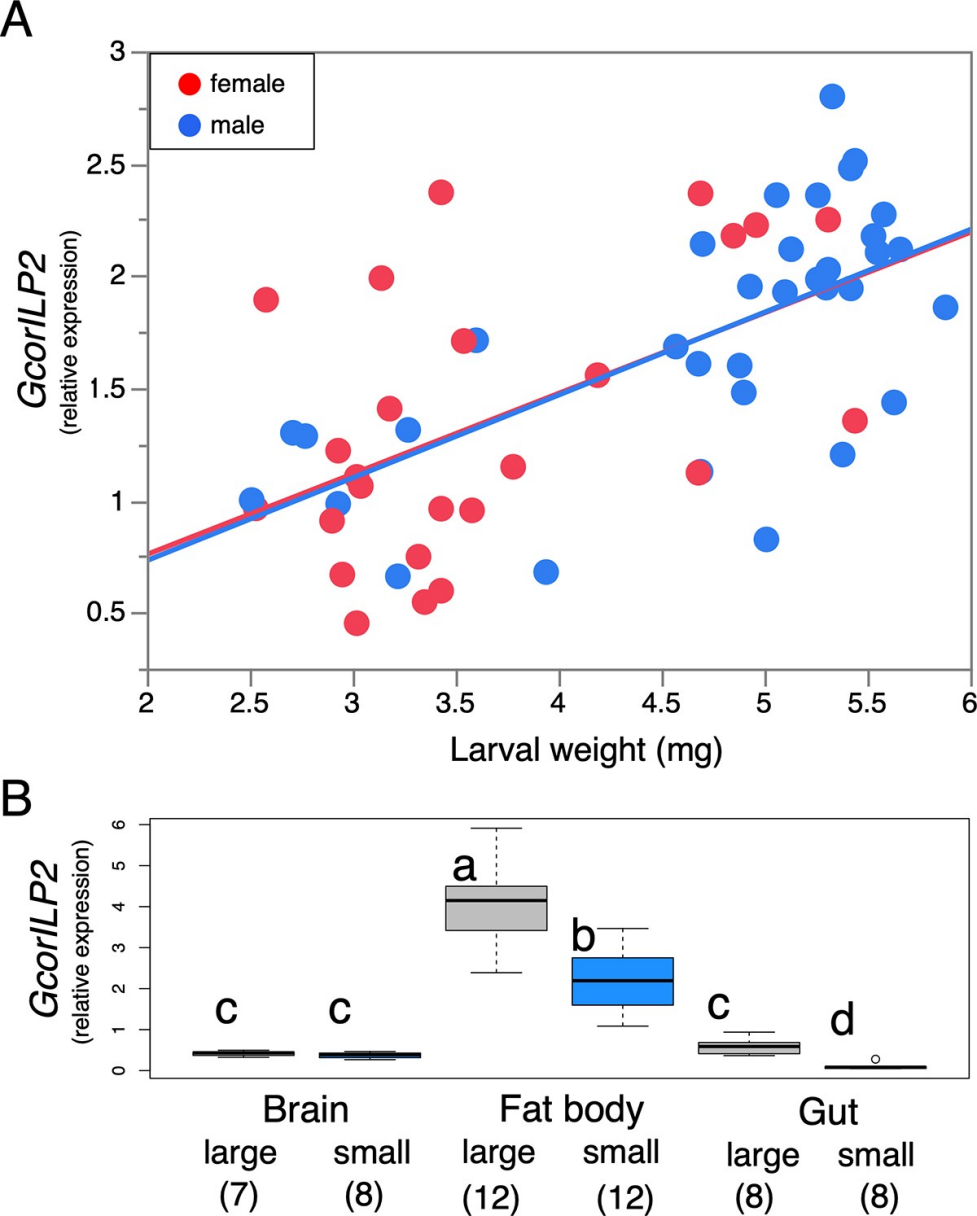
Levels of *GcorILP2* transcription in the fat body of larvae. (A) Both males (blue) and females (red) exhibited body size–dependent *ILP2* expression (regression analysis, female, Y = 0.359X + 0.040, R^2^ = 0.25, *p* = 0.01; male, Y = 0.369X − 0.006, R^2^ = 0.42, *p* < 0.0001). *n* = 25 and 34 for females and males, respectively. (B) *GcorILP2* was specific to the fat body and more abundant in large larvae. Different letters indicate significant differences (Steel-Dwass test). Gray (left): large; blue (right): small. Day-2 larvae were subjected to analysis. Gene expression levels relative to *Gcorgapdh* were quantified by qPCR. The underlying data in this figure are available from figshare (DOI: 10.6084/m9.figshare.9734780; https://figshare.com/s/609486022a3df39169bf). *Gcorgapdh*, *G*. *cornutus* glyceraldehyde 3-phosphate dehydrogenase; *GcorILP2*, *G*. *cornutus ILP2*; ILP2, insulin-like peptide 2; qPCR, quantitative PCR.

To further demonstrate the positive relationships between nutritional condition and *GcorILP2* expression, we additionally examined the conditional expression of *GcorILP2* in day-2 larvae with larval sexing and weighing. We found that there were significant positive relationships between larval weight and *GcorILP2* levels in both males and females (Fig 4A).

To determine the production source of GcorILPs, tissue-specific expression of *GcorILPs* was analyzed by qPCR using dissected brain, fat body, and gut tissues, which are the major sources of insect ILPs [18,44], in day-2 larvae. In insects, the principal ILP-producing cells that are tightly associated with nutrient-dependent growth and metabolism regulation are considered to be the neurosecretory cells in the brain [15]. The IGF-like ILPs in *Drosophila* (DILP6) and *Bombyx* (BIGFLP) are mainly produced in the fat body [33–35], and DILP7 is produced in neurons of abdominal ganglia [45,46]. Consistent with this, insulin-like *GcorILP1*, *GcorILP5*, and Dilp7-like *GcorILP4* were predominantly expressed in the brain but did not exhibit heightened expression in large larvae (S5 Fig).

IGF-like GcorILP3 was ubiquitously expressed across tissues, and its expression was heightened in the gut of large larvae but not in other tissues (S5 Fig). Interestingly, one of the ILPs, *GcorILP2*, was specifically and highly expressed in the fat body, and this expression was more abundant in large larvae (Fig 4B). During dissection, we noticed that the body cavity of large larvae is almost filled with the fat body, whereas that of small larvae contained less fat body (approximately half of the body volume, personal observation by YO). Therefore, the whole-body differences in *GcorILP2* expression (Fig 3B) may be explained by both fat body activity and the relative amount of fat body per individual. The above stage- and tissue-specific expression analyses pose a hypothesis that the fat body–derived GcorILP2 could be the best candidate for the molecular nature that regulates nutrient-dependent weapon growth in *G*. *cornutus*.

### GcorILP2-coupled condition with weapon growth

RNA interference (RNAi)-driven gene KDs were conducted to test the developmental functions of five *GcorILPs* and two *GcorInRs* by double-stranded RNA (dsRNA) injection in the final instar larvae. dsRNA dosages were gradually titrated down from 50 ng to 0.1 ng (50 ng, 10 ng, 1 ng, 0.2 ng, and 0.1 ng) to find the highest dosages providing viable adults that allowed for phenotyping (dosages and KD efficiencies are summarized in S1 Table).

**Fig 5:**
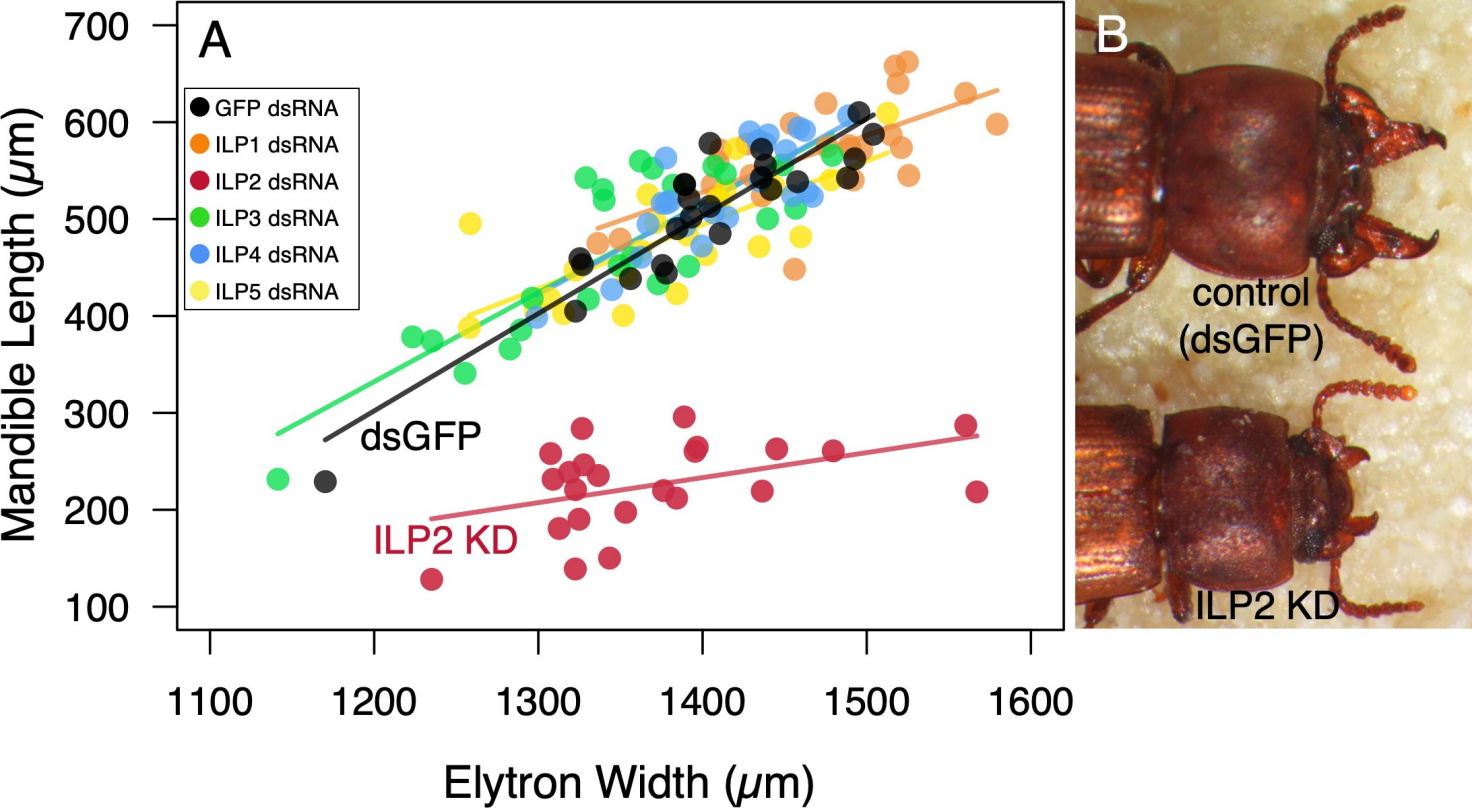
KD of a specific type of ILP (*GcorILP2*) diminishes conditional growth of male mandibles in *G*. *cornutus*. (A) KD phenotypes of 5 ILPs (*Gcor1-5*) shown as scatterplots of mandible length against body size (elytron width). Dots and regression lines of different treatments are shown by different colors. *n* = 24, 23, 24, 24, 24, and 24 for ds*Gcor1–5* and dsGFP, respectively. (B) *GcorILP2*^RNAi^ males had smaller mandible size than the control males had. Two males had similar elytra sizes but clearly different mandible sizes and moderately different head and prothorax sizes. The underlying data in this figure are available from figshare (DOI: 10.6084/m9.figshare.9734780; https://figshare.com/s/609486022a3df39169bf). dsGFP, double-stranded RNA for GFP; dsRNA, double-stranded RNA; *GcorILP2*, *G*. *cornutus* ILP2; GFP, green fluorescent protein; ILP, insulin-like peptide; KD, knockdown; RNAi, RNA interference.

We found that silencing *GcorILP2* (45% transcript reduction, S1 Table) yielded a clear reduction in male mandible size (Fig 5A and 5B). In *GcorILP2*^*RNAi*^ males, a clear decline of regression slope was detected compared to the control treatment (double-stranded RNA for green fluorescent protein [dsGFP]) (Fig 5A, ANCOVA [KD treatment as a factor, body size defined as elytra width {EW} as a covariate], body size × treatment, F = 26.1, *p* < 0.001, Bonferroni correction, see S2 Table for full model statistics), implying that body size–dependent growth of mandibles was diminished in *GcorILP2*^*RNAi*^ males.

In contrast, interaction terms (treatment × body size) were not significant in the other four *GcorILP* KDs (Fig 5A, ANCOVA, body size × treatment, *GcorILP1*^*RNAi*^, F = 6.43, *p* = 0.089; *GcorILP3*^*RNAi*^, F = 0.28, *p* = 1; *GcorILP4*^*RNAi*^, F = 0.132, *p* = 1; *GcorILP5*^*RNAi*^, F = 4.91, *p* = 1, Bonferroni correction). In these cases, the interaction term (body size × treatment) was removed from the statistical model. Consequently, the effects of KD treatments were not significant, indicating that regression intercepts were not altered by KDs of these ILPs (ANCOVA, treatment: *GcorILP1*^*RNAi*^, F = 0.346, *p* = 1; *GcorILP3*^*RNAi*^, F = 1.45, *p* = 1; *GcorILP4*^*RNAi*^, F = 1.71, *p* = 0.99; *GcorILP5*^*RNAi*^, F = 0.25, *p* = 1, see S2 Table for full model statistics).

Since *GcorILP* KDs are conducted at the highest levels (37%–85% reductions, S1 Table), morphogenetic effects of *GcorILP* KDs other than those of *GcorILP2* are negligible, at least at the prepupal stage. Additionally, log–log scale allometric analysis of mandible length against body size (defined as EW) showed that allometric coefficient α was >1 in the control treatment (α = 3.37 ± 0.65 [95% CI]) but was not different from 1 in *GcorILP2*^*RNAi*^ males (α = 1.86 ± 1.58 [95% CI]), supporting the positive allometry in control males but not in *GcorILP2*^*RNAi*^ males. These results strongly suggest that GcorILP2 is the major molecular nature that facilitates conditional growth of the weapon. Generally, KD of *GcorILPs* did not affect survival from larva to adult, and the eclosed adults exhibited seemingly normal phenotypes.

### GcorInR1 and GcorInR2 have functional redundancy for weapon morphogenesis

**Fig 6:**
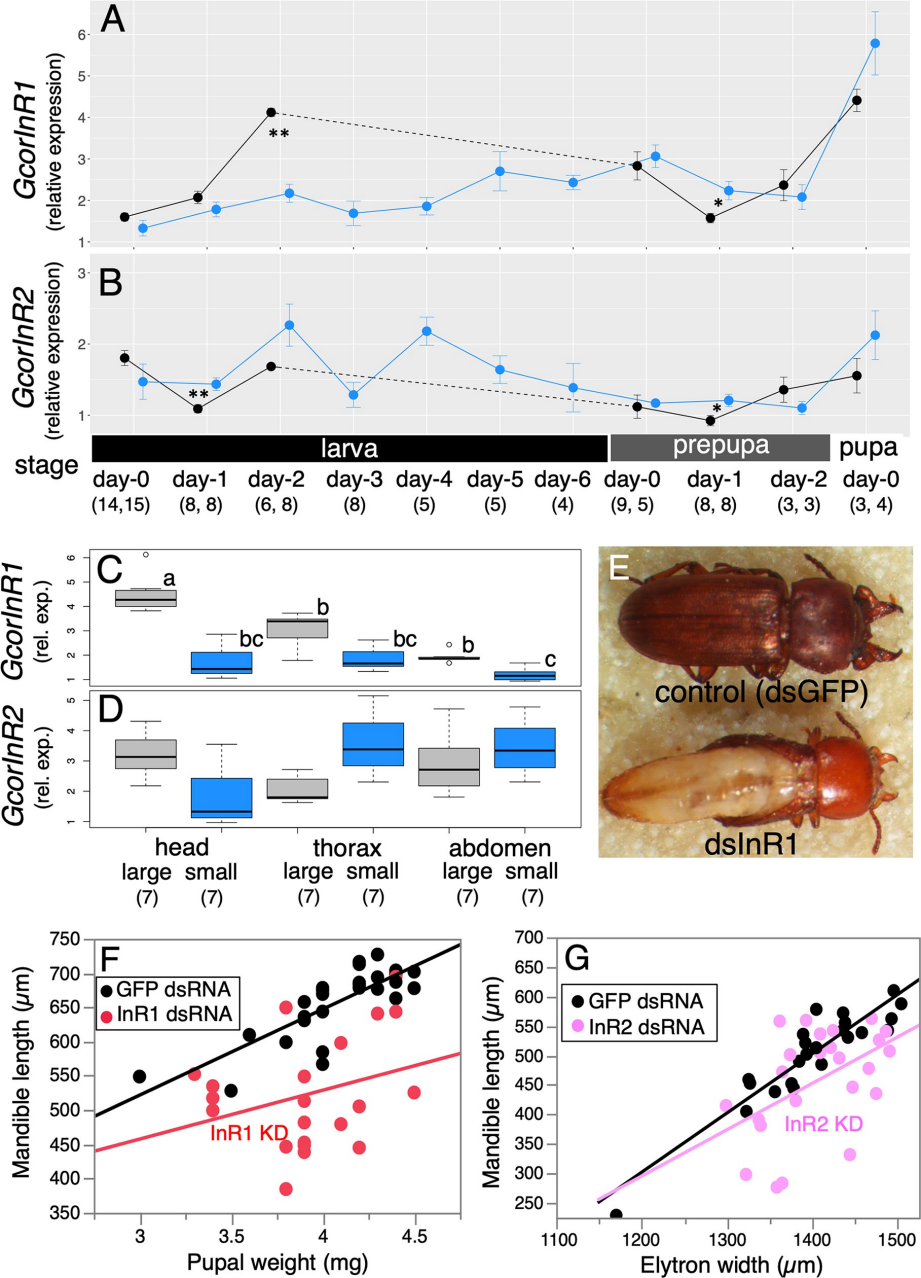
Functional analysis of insulin-like receptors. (A,B) Gcor*InR1* and Gcor*InR2* developmental dynamics, black: large larvae, blue: small larvae. Results are presented as mean ± SE. Asterisks show significant differences between large and small larvae of the same stages (Mann-Whitney U test, *p < 0.05, **p < 0.01, ***p < 0.001). See Fig 3 caption for abbreviations day-0, -1, etc. (C,D) Body-part specificities of *GcorInR1* and *GcorInR2*. Different letters indicate significant differences (Steel-Dwass test). Gray: large, blue: small. Day-2 larvae were subjected to analysis. Gene expression levels relative to *Gcorgapdh* were quantified by qPCR. (E) *GcorInR1*^RNAi^ individuals suffered from severe systemic defects characterized by the malformation of fore- and hind wings, even under the weakest KD (28% reduction, S1 Table). (F) Mandible lengths were plotted against pupal weights for teratogenic *GcorInR1*^RNAi^ males and normal dsGFP males (*n* = 21 and 29, respectively). (G) *GcorInR2*^RNAi^ males exhibited smaller mandible size without teratogenesis (*n* = 24 each) under intermediate level of KD (36% reduction, S1 Table). The underlying data in this figure are available from figshare (DOI: 10.6084/m9.figshare.9734780; https://figshare.com/s/609486022a3df39169bf). dsGFP, double-stranded RNA for GFP; dsRNA, double-stranded RNA; *Gcorgapdh*, *G*. *cornutus* glyceraldehyde 3-phosphate dehydrogenase; *GcorInR*, *G*. *cornutus* InR; GFP, green fluorescent protein; InR, insulin-like receptor; KD, knockdown; qPCR, quantitative PCR; rel. exp., relative expression; RNAi, RNA interference.

Next, we focused on the expression patterns and functions of two *GcorInRs* during development. The quantification of whole-body transcript levels from larva to pupa revealed that large individuals showed increased levels of *GcorInR1* during the larval stage (i.e., day-2 larvae), whereas these differences were diminished in prepupa day-0 and later stages (Fig 6A). *GcorInR2* expression showed no clear positive nutritional response during development (Fig 6A).

We further determined whether the expressions of two *GcorInRs* are body part specific, using the head, thorax, and abdomen of day-2 larvae. It is important to note that the head sample included mandible primordia and that the head itself exhibits positive allometry against body size [38,47]. We found that Gcor*InR1* was highly expressed in the head of the large larvae (Fig 6C). Heightened expression of *GcorInR1* was also observed in the thorax of large larvae, but it was not significant in the abdomen (Fig 6C). *GcorInR2* was ubiquitously expressed across body parts (Fig 6D). Therefore, conditional expression of Gcor*InR2* was unclear, although large larvae tended to express higher levels of *GcorInR2* in the head but nonsignificantly (Fig 6D).

Unlike *GcorILP*s, gene KD of *GcorInR1* was highly lethal before pupation. Even the smallest-dosage treatments (0.1 ng dsRNA, 28% reduction, S1 Table) caused the teratogenesis of adult posterior body and the failure of eclosion (see Fig 6E). This result is consistent with previous observations in *Drosophila* and *Tribolium* that *InR* mutant or KD phenotype showed severe defects in body growth and development [41,48]. Therefore, for *GcorInR1*^*RNAi*^ males, we used the pupal weight as an index of body size, and only adult mandibles were measured. *GcorInR1*^*RNAi*^ males exhibited a significant reduction of intercept (Fig 6F, ANCOVA, effect of treatment, F = 49.5, *p* < 0.001, see S2 Table for full model statistics). In contrast to *GcorInR1*^*RNAi*^ beetles, *GcorInR2*^*RNAi*^ beetles successfully eclosed under intermediate levels of KD (1 ng dsRNA, 36% reduction, S1 Table). In *GcorInR2*^*RNAi*^ males, there also was a significant decrease in mandible length as an intercept reduction (Fig 6G, ANCOVA, treatment, F = 8.43, *p* = 0.034, Bonferroni correction, see S2 Table for full model). Therefore, these two receptors likely have redundant functions in weapon growth. However, *GcorInR1* is more fundamental to systemic development, whereas *GcorInR2* is functionally more specific to mandible growth.

To quantify the changes in other traits, we firstly assessed the degree of trait size changes against dsGFP control in all treatments (S3 Table). Measurable *GcorInR1*^*RNAi*^ adults were not obtained; thus, they were excluded from the analysis. In *GcorILP2*^*RNAi*^ males, mandible length and width were greatly reduced (38%‒55%); horn, head, and prothoracic sizes were moderately reduced (9%‒25%); and elytra size was more consistent across treatments (2%‒4%), indicating that *GcorILP2* KD predominantly affects the mandibles but also reduces the anterior body size. Secondly, the change in overall phenotype was assessed by principal component analysis (PCA) using nine body-part measurements (S6 Fig, factor loadings in S4 Table). The lowered PC1 score of *GcorILP2*^*RNAi*^ males reflects the size reduction in anterior body parts. The lowered PC2 scores of *GcorILP2*^*RNAi*^ and *GcorInR2*^*RNAi*^ males indicated that KDs of these two genes yielded phenocopies similar in shape (i.e., relatively small anterior parts with larger posterior parts).

Taken together, these results strongly suggest that the fat body–derived GcorILP2 regulates nutrient-dependent weapon growth through two functionally redundant GcorInRs, one of which (GcorInR2) has partially gained a weapon-specific morphogenetic function in *G*. *cornutus*.

## Discussion

### Specific ILP mediates conditional growth of weapon

Our study revealed that one specific type of ILP, GcorILP2, positively correlated with nutritional condition and had a specific function in weapon growth. Interestingly, size-dependent mandible growth was diminished, and mandible length was nearly maintained at the smallest size under KD of ILP2. These results strongly support that ILP2 relays the nutritional condition to weapon growth in *G*. *cornutus*. So far, IIS has been proposed as the mechanism mediating conditional growth by receptor KD in horned beetles [10,12]. However, the exact ligand molecule that directly correlates with nutritional condition has not been identified in relation to the insect weapon. Besides insects, a rare example is found in deer, in which IGF-1 acts as a condition-dependent signal to accelerate antler growth [49,50]. Since IGF-1 is predominantly synthetized in the vertebrate liver, it is interesting to note that the liver and fat body, the two functionally equivalent organs between vertebrates and insects, have similar endocrinological systems to couple internal conditions with exaggerated trait growth. To our knowledge, this is the first evidence in insects to specify the actual messenger of IIS that underpins the conditional expression of sexually selected exaggerated traits.

In addition to the predominant effect on the mandibles, *GcorILP2* KD moderately reduced the head and prothorax sizes (S3 Table, S4 Table, S6 Fig). Such correlated changes in mandible and mandible-supporting traits are consistent with the developmental integration of functionally related modules (i.e., supportive traits, [51–53]). Unexpectedly, although similarly sized postfeeding larvae were subjected to KD experiments, the anterior body parts of *GcorILP2*^*RNAi*^ males decreased in size without increasing the other measured traits (S3 Table). We speculate that adult internal structures (e.g., fat body) are increased in *GcorILP2*^*RNAi*^ males, but this should be tested in future studies. Additionally, although genetic variation was considered to be low in our *G*. *cornutus* stock (see Materials and Methods), it may still contain some genetic variation. Thus, more work is required to determine whether genetic variation may also contribute to variation on larval growth dynamics.

### Diversification of insect ILPs and GcorILP2 function

From previous studies on *Drosophila*, ILP-mediated organ growth during larval development is generally considered to be a two-step process. The nutritional state is mainly sensed by the fat body [54], which in turn remotely regulates DILP secretion from brain neurosecretory cells through humoral signals called fat body–derived signals [55,56]. DILPs secreted from brain neurosecretory cells in turn control nutrient-dependent systemic body growth and metabolism during larval development. However, our results imply that the fat body–derived GcorILP2 is used as a direct signal for adult organ growth without intervening in the central neurosecretory regulation.

Given that holometabolous insect larvae accumulate lipid and protein in the fat body for metamorphic morphogenesis [57], it is reasonable to assume that the larval fat body harbors information of nutritional state. At the postfeeding metamorphic stage, condition-dependent synthesis of GcorILP2 occurs in the fat body (peaking in day-2 larvae; Fig 3B) to regulate adult mandible growth in broad-horned flour beetles. Therefore, we suggest that, unlike the aforementioned two-step process, *G*. *cornutus* has a simple but elaborate growth regulatory mechanism in which a specific type of fat body–derived ILP directly couples nutritional condition with weapon growth. Whether the homologous and/or fat body–derived ILP subtype is repeatedly deployed in the conditional growth of weapons in other beetles and insects that independently gain weapons is an especially intriguing evolutionary question for future studies.

Importantly, however, our current argument on insect ILPs is largely based on derived holometabolous insects, such as Diptera and Lepidoptera. Therefore, the generality of the aforementioned two-step process is still uncertain, and the evolutionary process of insect insulin signaling is open to debate.

Our findings reflect the functional diversification of ILPs in other insect species. For example, ovarian activity is specifically regulated by *A*. *aegypti* ILP3 (*AaILP3*) in a mosquito and *Ooceraea biroi* ILP2 (*ObILP2*) in a clonal ant [23,58]. In body growth and metabolism, *AaILP6* has a specific interactive function with serotonin signaling in the fat body [25]. Combined with these previous studies, our findings highlight that ILP diversification plays a fundamental role in the ecophysiological diversification of insects. However, future comparative physiological studies across taxa are essential to understand the evolution of insect IIS.

### Implication for module-specific complex growth

By receptor KDs, *GcorInR1*^*RNAi*^ males suffered high lethality, whereas *GcorInR2*^*RNAi*^ males could survive to become normal adults, suggesting that GcorInR1 plays highly pleiotropic roles in systemic development (Fig 6E). A similar deleterious effect of InR1 KD was also reported in red flour beetles during metamorphosis [41]. In contrast, relatively strong silencing of *GcorInR2* (36%) yielded normal adults with reduced mandibles (Fig 6G, S6 Fig), implying that, although GcorInR1 and GcorInR2 are functionally redundant in mandible development, GcorInR2 has a more specific effect on mandible growth.

We would expect that InR localization can explain weapon-specific growth patterns [59]; however, the localization of *GcorInR1* and *GcorInR2* was paradoxical. *GcorInR1*, which had systemic effects, was strongly expressed in the head, whereas InR2, which had a specific effect on the mandibles, was ubiquitously expressed in the whole body (Fig 6C and 6D). An additional analysis confirmed that KD efficiency of GcorInR2 was similar across body parts (43%, 41%, and 48% for the head, thorax, and abdomen, respectively, S1 Table), although there was a slight interaction effect between body part and KD treatment (two-way ANOVA, body part × treatment, F = 3.58, *p* = 0.037). Therefore, the module specificity of KD effect and localization of *GcorInR2* did not coincide, and the aforementioned receptor-localization hypothesis was only partly supported for *GcorInR1*. We consider that the pleiotropic negative effect of *GcorInR1* was avoided by its localized expression, whereas *GcorInR2* somehow gained a weapon-specific growth function.

Theories of evolutionary genetics propose that duplicated genes with functional redundancy often differentiate into a state in which one of them gains functional novelty (i.e., subfunctionalization to neofunctionalization) [36,37,60]. A recent study in a dung beetle proposed a potential interaction of *doublesex* (dsx, the regulator of sex differentiation) with *InR2* but not with *InR1*, implying the functional differentiation of the two InRs [12]. The partly overlapping but differentiated functions of the two GcorInRs, as well as the distinctive weapon-specific function of GcorILP2, can be understood in such evolutionary process, i.e., the functional diversification following the gene duplication.

As for module-specific conditional growth, several other mechanisms have been proposed (e.g., FOXO [12,61,62]; HDAC [42]). Additionally, juvenile hormone (JH), the central regulator of insect metamorphosis, is another well-known factor that facilitates weapon growth in several species, including *G*. *cornutus* [53,63–65]. Interestingly, *GcorILP2* ortholog *TcILP2* expression is inducible by JH in adult red flour beetles [66]. Future research to elucidate the links between these molecular pathways, as well as the downstream action of IIS and combinatorial functions of different ILPs, is required to fully understand the mechanisms underlying the complex growth of secondary sexual traits.

In conclusion, our study illustrates that functionally diversified IIS genes underlie the evolution of complex growth regulation in exaggerated traits.

## Materials and methods

### Broad-horned flour beetle *(G*. *cornutus)*

The *G*. *cornutus* stock population originated from adults collected in Miyazaki City, Japan. They were reared in the National Food Research Institute, Japan, and Okayama University, Japan, for about 50 years [38]. Therefore, the genetic variation in the stock population may be low. The stock was maintained with wholemeal flour enriched with yeast. The stock populations were reared in plastic containers (diameter, 40 mm; height, 30 mm) in groups of approximately 200 larvae, and they were provided with sufficient culture medium (wholemeal enriched with brewer’s yeast [EBIOS, Asahi Group Foods, Tokyo, Japan]) [67].

### Developmental schedule and staging

Timing of pupation is determined through critical size and food availability in *G*. *cornutus*. Additionally, its pupation is inhibited by larval crowding to avoid being the victim of cannibalism during molting, as is generally the case for Tenebrionidae [67,68]. In our experiment, larvae aged from 30 to 60 days after hatching (approximately 2‒6 mg in weight) were transferred from sufficiently fed culture conditions to unfed solitary conditions (individual wells in a 24-well plate, VTC-P24, VIOLAMO) (Fig 1) [68]. We set these criteria according to the previous description and preliminary experiment that showed that they take approximately 30 days to reach the smallest size and 60 days to reach the maximum size [39,69].

The isolation from the stock (i.e., isolation from high-density conditions and food) led them to pupate within about a week; therefore, the age at isolation approximates the size of the focal larva and, consequently, that of the developed adult in this species. In Tenebrionidae, the number of instars and developmental time can vary according to diet conditions [70]. In our procedure, the total amount of diet was experimentally manipulated by isolating the larvae from the stock at various sizes and timings. In this procedure, the larvae that were <2.5 mg frequently failed metamorphosis and eventually died; therefore, we considered 2.5 mg to be the smallest size that allowed them to survive and develop into adults (i.e., critical size). Using the median size of larvae capable of metamorphosis as a criterion (S1 Fig), the larvae weighing 2.5‒4 mg were defined as “small individuals” with poor nutrition and those weighing >4 mg as “large individuals” with abundant nutrition. To detect the size difference, we used the largest larvae (>4.5 mg) for the large category when available (85% [44 of 52] of large individuals were >4.5 mg).

The larval stages were defined as the days after isolation (e.g., a larva aged 1 day after isolation is referred to as a day-1 larva). Prepupation was visually checked every 24 hours to detect their characteristic L-shaped posture [67] (Fig 1). After the isolation from the stock, large larvae (>4 mg) soon proceeded to the prepupal stage (2‒4 days, Fig 1, S1 Fig), whereas small larvae (<4 mg) took longer for prepupation (3‒8 days).

### Developmental dynamics and tissue specificities of insulin signaling genes

Transcript dynamics from larval to pupal stages were examined by qRT-PCR using the whole body. For whole-body samples, total RNA was extracted with TRIzol (Invitrogen), treated with DNAse I (RNA aqueous Micro Kit, Ambion), and reverse transcribed following the manufacturer’s protocols of the High capacity cDNA RT kit (Applied Biosystems). Kapa SYBR Fast qPCR kit (KAPA) and Thermal Cycler Dice Real Time System II (Takara) were used to conduct qPCR, with gene-specific primers (S3 Table), fast PCR protocols, and crossing-point method following the manufacturer’s instructions (Takara). According to the preliminary experiment, *Gcorgapdh* was confirmed as an appropriate normalization gene and thus used as a control gene in qRT-PCR. Relative quantification with the standard curve method was applied. Note that large larvae take less developmental time for prepupation (Fig 1, S1 Fig), and therefore, day-3 to day-6 large larvae were not available (Figs 2 and 6, dashed line).

To analyze body-part specificities of transcripts (Fig 6C and 6D), day-2 larvae after isolation were dissected in ice-cold 1 × PBS buffer. Individual larvae were used as cDNA samples of the head, thorax, and abdomen (*n* = 7 individuals). For small tissues (Fig 4B, S5 Fig), eight individuals were lumped together to obtain brain, fat body, and gut cDNA samples, and this procedure was replicated 7–12 times. For these tissue samples, total RNA was extracted with RNA aqueous Micro Kit (Ambion) and subjected to qPCR as described above.

For larval sexing (Fig 4), dsx primers spanning the sex-specific splicing region were used (S5 Table). The cDNA of test larvae was subjected to PCR (Takara exTaq, 35 cycles, 94°C for 1 minutes, 55°C for 30 seconds, and 72°C for 30 seconds). PCR products were subjected to agarose gel electrophoresis, and sex-specific fragment patterns were used for sexing [71]. Individuals used in this analysis (Fig 4) partly overlapped with those used in Fig 3 as day-2 larvae (*n* = 14).

### KDs of insulin signaling genes

Fully grown final instar larvae (approximately 60 days after hatching) were randomly selected from the stock and subjected to the dsRNA injection using Nanoject II (Drummond Scientific) under CO_2_ anesthesia, then kept individually in 24-well plates without food. When KD efficiency was too severe to kill the test larvae and/or yielded teratogenic adults unsuitable for morphological measurements, dsRNA dosages were gradually titrated down from 50 ng to 0.1 ng (50 ng, 10 ng, 1 ng, 0.2 ng, and 0.1 ng). Among these, we selected the highest doses that allowed normal adult emergence (S1 Table).

Obtained adults (randomly selected 23‒24 males for all treatments) were measured using a dissection microscope (VHX-200; KEYENCE) for mandible length, mandible width, horn length, gena width (lateral head structure), frontal prothorax width, maximum prothorax width, prothorax length, elytron length, and elytron width, as in previously described methods [42]. Elytron width was used as the index of body size [42]. As a control treatment, dsGFP (1 ng) was injected and analyzed as above. Bonferroni correction was applied to control for the multiple comparisons against the control. KDs of some genes were deleterious, and normal adults were not available even at the lowest levels of KDs (0.1 ng of dsRNA). In these cases, pharate adults were measured, and pupal weight was used as the body size.

### Identification and classification of ILPs and InRs

Using the four *ILP*s (*ILP1–4*) [28] and two putative ILP receptors from the red flour beetle (InR1 and InR2, Gene IDs: 661524 and 664271) as queries, *G*. *cornutus ILP* and *InR* candidates were retrieved by local BLASTx against the larval transcriptome (e-value < 1 for ILPs, e-value < 0.0001 for InRs) [42], and then the reciprocal best BLAST hit was confirmed [72]. Obtained sequences were manually checked for their identities by constructing amino acid alignments and phylogeny as follows.

For the ILP protein tree, annotated ILPs in flour beetles (*n* = 4) were included in the analysis. For the InR protein tree, we additionally used three coleopteran and four noncoleopteran insects with genome or transcriptome information available and human InR and IGF1R (S4 Fig) for speculation of cross-species orthologies. The evolutionary history was inferred by the maximum-likelihood method with a JTT matrix-based model and evaluated by bootstrap analyses (*n* = 1,000) using MEGA7 [73]. Analytical details are provided in figure captions (S3 and S4 Figs).

Owing to the extraordinarily diverged amino acid sequences in ILPs [34], ILP amino acid sequences were manually aligned by focusing on domain structures and characteristic cysteine residues according to a previous study [18].

### Statistical analysis

For gene expression across tissues, either a parametric (Tukey’s HSD) or nonparametric (Steel-Dwass test) method was applied according to the assessment of data distribution (Shapiro-Wilk test, *p* > 0.05, and Levene’s test, *p* > 0.05 for parametric test). For the comparison of KD and control phenotypes, we used ordinary least-square regression and ANCOVA to analyze the effect of gene KD on mandible length using elytra length as a covariate (mandible length = elytra length + KD treatment + elytra length × KD treatment). When the interaction term (elytra length × KD treatment) was not significant after Bonferroni correction, the interaction term was removed from the model. We used EW as an index of body size because EW was the most stable trait across treatments (S4 Table). The statistical analyses were performed by JMP11 (SAS Institute) and R 3.5.1. (R Core Team 2018).

## Supporting information

S1 Table dsRNA dosages and RNAi efficiencies. dsRNA, double-stranded RNA; RNAi, RNA interference.
(DOCX)

S2 Table Effect of gene KD on mandible length. Mandible lengths of KD treatments were compared to dsGFP control by ANCOVA using body size as covariate. Statistically insignificant interaction terms (*p* > 0.05) were removed from the model. For *GcorILP1-5* KD and *GcorInR2* KD, elytra width was used as body size. Pupal body weight was used as body size for *GcorInR1* KD in which measurable adults did not eclose (also see Results). For multiple comparison of *GcorILP1-5* KD and *GcorInR2* KD to the control treatment, *p*-values were adjusted by Bonferroni correction. dsGFP, double-stranded RNA for green fluorescent protein; *GcorILP1-5*, *G*. *cornutus* insulin-like peptides 1–5; *GcorInR2*, *G*. *cornutus* insulin-like receptor 2; KD, knockdown.
(DOCX)

S3 Table Effect of gene KD on absolute trait size. Mean ± SE is shown (μm). Fold changes to dsGFP control in mean sizes were shown in parenthesis. Bold letters indicate statistically significant differences from the control (Dunnett’s test, *p* < 0.05). Note that mandibular traits are greatly reduced, head and thoracic traits are moderately reduced, and elytron traits are consistent by GcorILP2 KD. dsGFP, double-stranded RNA for green fluorescent protein; EL, elytra length; EW, elytra width; FPW, frontal prothorax width; GcorILP2, *G*. *cornutus* insulin-like peptide 2; GW, gena width; HL, horn length; KD, knockdown; ML, mandible length; MPW, maximum prothorax width; MW, mandible width; PL, prothorax length.
(DOCX)

S4 Table Factor loadings for principal component analysis. (DOCX)

S5 Table Primer sequences for dsRNA synthesis and qPCR. *primers for sexing. dsRNA, double-stranded RNA; qPCR, quantitative PCR.
(DOCX)

S1 Fig Developmental schedule depends on larval body size. The smaller larvae took longer time for prepupation after isolation from stock culture (i.e., high density and abundant food). Male, Y = −1.175X + 9.13, R^2^ = 0.50, *p* < 0.001. Female, Y = −1.950X + 11.7, R^2^ = 0.60, *p* < 0.001 (regression analysis).
(DOCX)

S2 Fig Predicted prepropeptide structures of insulin-like, IGF-like, and DILP7-like peptides in *G. cornutus*. Domain-based alignment of (A) insulin-like peptides, (B) IGF-like peptides, and (C) DILP7-like peptides from broad-horned flour beetle (*G*. *cornutus*), red flour beetle (*T*. *castaneum*), fruit fly (*D*. *melanogaster*), yellow fever mosquito (*A*. *aegypti*), and honey bee (*A*. *mellifera*). Representative ILPs of *D*. *melanogaster* (DILP2, -6, -7), *A*. *aegypti* (AaegILP3, -5, -6), and *A*. *mellifera* (AmILP1, -2) were shown. Highly conserved amino acid residues between all ILPs are shown in red, and highly conserved amino acid residues between orthologous ILPs in *G*. *cornutus* and *T*. *castaneum* are shown in green. Color bars indicate the predicted domains in the precursor peptides: green, signal peptide; red, B-chain; yellow, C-peptide; blue, A-chain; gray, D-domain. Asterisks on the color bars below the alignment denote Cys residues, and paired triangles denote potential cleavage sites (dibasic amino acids). GcorILP1-4 showed orthologous relationship with TcILP1-4, whereas GcorILP5 has no clear ortholog in *T*. *castaneum*. A group of insulin-like peptides (A) shares the most common structural feature of the ILP family, and GcorILP1, -2, -5 are classified into this group. The common feature of this group is a conserved domain organization of their precursors, consisting of a signal peptide, with a B-chain, C-peptide, and A-chain. After cleavage of the signal peptide, the C-peptide is most likely removed to generate a mature heterodimeric peptide consisting of the A- and B-chains like vertebrate insulin. A group of IGF-like peptides (B) is characterized by a relatively shortened or truncated C-peptide like vertebrate IGFs, and GcorILP3 is classified into this group. GcorILP3 has an extended A peptide (D-domain) as seen in TcILP3 and AaegILP6 [30], which are more like the vertebrate IGFs. The third group, DILP7-like peptides (C), is characterized by an unusually conserved sequence shared by several insects, and GcorILP4 is classified into this group. AaegILP, *A*. *aegypti* ILP; AmILP, *A*. *mellifera* ILP; DILP, *Drosophila* ILP; GcorILP, *G*. *cornutus* ILP; IGF, insulin-like growth factor; ILP, insulin-like peptide; TcILP, *T*. *castaneum* ILP.
(DOCX)

S3 Fig Insulin-like peptide phylogeny based on amino acid sequences. Four of five *GcorILP* sequences had close similarities with corresponding *TcasILP*s, suggesting the orthologous relationships of these four genes. Bootstrap values (%, *n* = 1,000, maximum likelihood) are shown on the branches. Partial deletion model (90%) with 84 positions were used in final dataset. *GcorILP*, *G*. *cornutus* insulin-like peptide; *TcasILP*, *T*. *castaneum* insulin-like peptide.
(DOCX)

S4 Fig Protein phylogeny of insulin-like peptide and IGF receptors. There are two major two clades in coleopteran InRs (type 1 and type 2 InRs). Twenty amino acid sequences from nine insect species and human were included in the analysis (abbreviations: first letter of genus and first three letters of species, *G*. *cornutus*, broad-horned flour beetle; *T*. *castaneum*, red flour beetle; *Leptinotarsa decemlineata*, Colorado potato beetle; *O*. *taurus*, dung beetle; *Nicrophorus vespilloides*, burying beetle; *Zootermopsis nevadensis*, dampwood termite; *A*. *mellifera*, honey bee; *D*. *melanogaster*, fruit fly; *Nilaparvata lugens*, brown planthopper; *Homo sapiens*, human). Bootstrap values (%, *n* = 1,000, maximum likelihood) are shown on the branches. Partial deletion model (90%) with 1,086 positions were used in final dataset. IGF, insulin-like growth factor; InR, insulin-like receptor.
(DOCX)

S5 Fig Source of *GcorILP1-5* transcripts. *GcorILP1*,*4*,*5* were predominantly synthetized in brain. *GcorILP1* expression was more abundant in small larvae. GcorILP3 was ubiquitously expressed across tissues. Different letters indicate significant differences (*GcorILP1*,*3*,*5*: Steel-Dwass test; *GcorILP4*: Tukey’s HSD test). Gray: large, blue: small. Day-2 larvae were subjected to analysis. Gene expression levels relative to *Gcorgapdh* were quantified by qPCR. *GcorILP*, *G*. *cornutus* insulin-like peptide; *Gcorgapdh*, *G*. *cornutus* glyceraldehyde 3-phosphate dehydrogenase; qPCR, quantitative PCR.
(DOCX)

S6 Fig Principal component analysis based on sizes of nine body parts. RNAi phenotypes were represented by PC1-PC2 plot. PC1 was a size component positively loaded by all traits, and PC2 was a shape component positively loaded by mandible length, mandible width, and horn length (see S2 Table for factor loadings). Reduced PC2 scores in ILP2^RNAi^ and InR2^RNAi^. ILP2, insulin-like peptide 2; InR2, insulin-like receptor 2; PC, principal component; RNAi, RNA interference.
(DOCX)

S1 Material Nucleotide sequences of *GcorILP*s and *GcorInR*s. *GcorILP*, *G*. *cornutus* insulin-like peptide; *GcorInR*, *G*. *cornutus* insulin-like receptor.
(DOCX)

## Abbreviations

AaILP3: *A*. *aegypti* ILP3
BIGFLP: IGF-like ILP in *Bombyx*
DILP: *Drosophila* ILP
dsGFP: double-stranded RNA for green fluorescent protein
dsRNA: double-stranded RNA
dsx: doublesex
EW: elytra width
GcorILP: *G*. *cornutus* ILP
GcorInR: *G*. *cornutus* InR
IGF: insulin-like growth factor
IIS: insulin/IGF signaling
ILP: insulin-like peptide
InR: insulin-like receptor
JH: juvenile hormone
KD: knockdown
ObILP2: *Ooceraea biroi* ILP2
PCA: principal component analysis
rel. exp.: relative expression
RNAi: RNA interference
TcILP: *T*. *castaneum* ILP
TcInR: *T*. *castaneum* InR.
