# Supplementary material for: A specific type of insulin-like peptide regulates the conditional growth of a beetle weapon
Source: PLoS Biol. 2019 Nov 27;17(11):e3000541. doi: 10.1371/journal.pbio.3000541 (PMC6880982; doi:10.1371/journal.pbio.3000541)
Supplement: S1 Table — dsRNA, double-stranded RNA; RNAi, RNA interference. (DOCX) [file pbio.3000541.s001.docx]

**S1 Table** dsRNA dosages and RNAi efficiencies

| target | dsRNA amount | KD efficiency (% reduction) | transcript level (control, 1ng GFP) | transcript level (RNAi) | *p* value (Student's *t*-test) |
| --- | --- | --- | --- | --- | --- |
| *GcorILP1* | 50ng | 52% | 0.327±0.032 (12) | 0.157±0.021 (7) | 0.0008 |
| *GcorILP2* | 50ng | 45% | 1.12±0.036 (12) | 0.62±0.040 (12) | <.0001 |
| *GcorILP3* | 50ng | 52% | 1.04±0.108 (12) | 0.54±0.032 (12) | 0.0001 |
| *GcorILP4* | 50ng | 85% | 1.94±0.197 (12) | 0.32±0.017 (12) | 0.0001 |
| *GcorILP5* | 50ng | 37% | 0.310±0.032 (12) | 0.195±0.014 (10) | 0.0035 |
|  |  |  |  |  |  |
| *GcorInR1* | 0.1ng | 28% | 4.51±0.195 (12) | 3.24±0.116 (12) | <.0001 |
| *GcorInR2* | 1ng | 36% | 1.95±0.132 (12) | 1.25±0.147 (12) | 0.0006 |
|  |  |  |  |  |  |
| *GcorInR2* |  |  |  |  |  |
| head | 1ng | 43% | 1.579±0.038 (8) | 0.904±0.063 (8) | <.0001 |
| thorax | 1ng | 41% | 1.131±0.050 (8) | 0.670±0.024 (8) | <.0001 |
| abdomen | 1ng | 48% | 1.203±0.013 (8) | 0.627±0.029 (8) | <.0001 |
