# Supplementary material for: A specific type of insulin-like peptide regulates the conditional growth of a beetle weapon
Source: PLoS Biol. 2019 Nov 27;17(11):e3000541. doi: 10.1371/journal.pbio.3000541 (PMC6880982; doi:10.1371/journal.pbio.3000541)
Supplement: S2 Table — Mandible lengths of KD treatments were compared to dsGFP control by ANCOVA using body size as covariate. Statistically insignificant interaction terms (p > 0.05) were removed from the model. For GcorILP1-5 KD and GcorInR2 KD, elytra width was used as body size. Pupal body weight was used as body size for GcorInR1 KD in which measurable adults did not eclose (also see Results). For multiple comparison of GcorILP1-5 KD and GcorInR2 KD to the control treatment, p-values were adjusted by Bonferroni correction. dsGFP, double-stranded RNA for green fluorescent protein; GcorILP1-5, G. cornutus insulin-like peptides 1–5; GcorInR2, G. cornutus insulin-like receptor 2; KD, knockdown. (DOCX) [file pbio.3000541.s002.docx]

**S2 Table** Effect of gene KD on mandible length

Mandible lengths of KD treatments were compared to dsGFP control by ANCOVA using body size as covariate. Statistically insignificant interaction terms (p>0.05) were removed from the model. For *GcorILP1-5* KD and *GcorInR2* KD, elytra width was used as body size. Pupal body weight was used as body size for *GcorInR1* KD in which measurable adults did not eclose (also see Results). For multiple comparison of *GcorILP1-5* KD and *GcorInR2* KD to the control treatment, p-values were adjusted by Bonferroni correction.

| gene KD | factor | F | p |
| --- | --- | --- | --- |
| *GcorILP1* | treatment | 0.35 | 1 |
|  | body size | 91.96 | <.001 |
|  | treatment x body size | - | - |
|  |  |  |  |
| *GcorILP2* | treatment | 74.74 | <.001 |
|  | body size | 553.83 | <.001 |
|  | treatment x body size | 26.13 | <.001 |
|  |  |  |  |
| *GcorILP3* | treatment | 1.45 | 1 |
|  | body size | 147.59 | <.001 |
|  | treatment x body size | - | - |
|  |  |  |  |
| *GcorILP4* | treatment | 1.71 | 0.99 |
|  | body size | 154.03 | <.001 |
|  | treatment x body size | - | - |
|  |  |  |  |
| *GcorILP5* | treatment | 0.25 | 1 |
|  | body size | 108.49 | <.001 |
|  | treatment x body size | - | - |
|  |  |  |  |
| *GcorInR1* | treatment | 49.48 | <.001 |
|  | body size | 17.72 | <.001 |
|  | treatment x body size | - | - |
|  |  |  |  |
| *GcorInR2* | treatment | 8.43 | 0.0342 |
|  | body size | 44.06 | <.001 |
|  | treatment x body size | - | - |
