# Supplementary material for: A specific type of insulin-like peptide regulates the conditional growth of a beetle weapon
Source: PLoS Biol. 2019 Nov 27;17(11):e3000541. doi: 10.1371/journal.pbio.3000541 (PMC6880982; doi:10.1371/journal.pbio.3000541)
Supplement: S3 Table — Mean ± SE is shown (μm). Fold changes to dsGFP control in mean sizes were shown in parenthesis. Bold letters indicate statistically significant differences from the control (Dunnett’s test, p < 0.05). Note that mandibular traits are greatly reduced, head and thoracic traits are moderately reduced, and elytron traits are consistent by GcorILP2 KD. dsGFP, double-stranded RNA for green fluorescent protein; EL, elytra length; EW, elytra width; FPW, frontal prothorax width; GcorILP2, G. cornutus insulin-like peptide 2; GW, gena width; HL, horn length; KD, knockdown; ML, mandible length; MPW, maximum prothorax width; MW, mandible width; PL, prothorax length. (DOCX) [file pbio.3000541.s003.docx]

**S3 Table** Effect of gene KD on absolute trait size.

Mean ± SE is shown (µm). Fold changes to dsGFP control in mean sizes were shown in parenthesis. Bold letters indicate statistically significant differences from the control (Dunnett’s test, p<0.05). Note that mandibular traits are greatly reduced, head and thoracic traits are moderately reduced, and elytron traits are consistent by GcorILP2 KD. Abbreviations: ML (mandible length), MW (mandible width), HL (horn length), GW (gena width), FPW (frontal prothorax width), MPW (maximum prothorax width), PL (prothorax length), EW (elytra width), EL (elytra length).

| KD treatment | n | ML | MW | HL | GW | FPW | MPW | PL | EW | EL |
| --- | --- | --- | --- | --- | --- | --- | --- | --- | --- | --- |
| *GFP* | 24 | 503±16 | 277±6 | 200±4 | 1364±24 | 1240±17 | 1516±18 | 1089±14 | 1400±15 | 1290±11 |
| *GcorILP1* | 24 | **569±11** (1.13) | 283±4 (1.02) | 204±5 (1.02) | **1451±18** (1.06) | 1283±13 (1.03) | **1583±15** (1.04) | **1139±10** (1.05) | **1471±12** (1.05) | 1331±11 (1.03) |
| *GcorILP2* | 23 | **226±10** (0.45) | **171±4** (0.62) | **168±4** (0.84) | **1024±12** (0.75) | **1123±10** (0.91) | **1401±12** (0.92) | **994±8** (0.91) | 1373±17 (0.98) | **1235±11** (0.96) |
| *GcorILP3* | 24 | 466±18 (0.93) | 259±6 (0.93) | 188±5 (0.94) | 1293±28 (0.95) | 1190±17 (0.96) | 1463±21 (0.97) | 1044±17 (0.96) | **1345±17** (0.96) | **1242±17** (0.96) |
| *GcorILP4* | 24 | 528±11 (1.05) | 278±4 (1.00) | 210±2 (1.05) | 1397±15 (1.02) | 1257±11 (1.01) | 1542±12 (1.02) | 1112±9 (1.02) | 1413±9 (1.01) | 1296±9 (1.00) |
| *GcorILP5* | 24 | 482±12 (0.96) | 277±4 (1.00) | 199±4 (1.00) | 1343±21 (0.98) | 1236±18 (1.00) | 1485±24 (0.98) | 1086±19 (1.00) | 1382±14 (0.99) | 1265±12 (0.98) |
| *GcorInR2* | 24 | 458±18 (0.91) | **218±6** (0.79) | 184±6 (0.92) | 1344±18 (0.98) | 1272±11 (1.03) | 1519±14 (1.00) | 1117±10 (1.03) | 1406±11 (1.00) | 1318±9 (1.02) |
