# Supplementary material for: A specific type of insulin-like peptide regulates the conditional growth of a beetle weapon
Source: PLoS Biol. 2019 Nov 27;17(11):e3000541. doi: 10.1371/journal.pbio.3000541 (PMC6880982; doi:10.1371/journal.pbio.3000541)
Supplement: S4 Table — (DOCX) [file pbio.3000541.s004.docx]

**Table S4** Factor loadings for principal component analysis.

|  | PC1(75.1%) | PC2(10.6%) |
| --- | --- | --- |
| ML (mandible length) | 0.877 | 0.343 |
| MW (mandible width) | 0.801 | 0.509 |
| HL (horn length) | 0.702 | 0.390 |
| GW (gena width) | 0.964 | 0.153 |
| FPW (frontal prothorax width) | 0.909 | -0.164 |
| MPW (maximum prothorax width) | 0.926 | -0.172 |
| PL (prothorax length) | 0.903 | -0.159 |
| EW (elytra width) | 0.832 | -0.404 |
| EL (elytra length) | 0.860 | -0.397 |
