# Supplementary material for: A specific type of insulin-like peptide regulates the conditional growth of a beetle weapon
Source: PLoS Biol. 2019 Nov 27;17(11):e3000541. doi: 10.1371/journal.pbio.3000541 (PMC6880982; doi:10.1371/journal.pbio.3000541)
Supplement: S5 Table — *primers for sexing. dsRNA, double-stranded RNA; qPCR, quantitative PCR. (DOCX) [file pbio.3000541.s005.docx]

**S5 Table** Primer sequences for dsRNA synthesis and qPCR

*primers for sexing

| Gene | RNAi primer ID | RNAi primer sequence | qPCR primer ID | qPCR primer sequence |
| --- | --- | --- | --- | --- |
| *GcorILP1* | T7GnaILPb54-287LEFT | TCTTCCCCTCACTTGACCCA | GnaILPb_q_304_373F | AGGAAAGCTGGAATCGTCGA |
|  | T7GnaILPb54-287RIGHT | ACAAACGAGTTCGCCGTTTC | GnaILPb_q_304_373R | CGCAGTACAAGCTGAGGTGT |
| *GcorILP2* | T7GnaILPa6-221LEFT | TCTTCAGTGCGTCTTCGTGG | ILPa-qPCR-271-366F | ATGACAAACTTTCGGCGTCG |
|  | T7GnaILPa6-221RIGHT | ATAAACTGGCCTTGGCTCCC | ILPa-qPCR-271-366R | GCTGCCGCAGTACGTTTTTA |
| *GcorILP3* | T7GnaILPd39LEFT | GGTGTCCTTGTTCGCTCTCA | GnaILPd_q_210-422F | TACAAAAGAGCCAGTCCCGG |
|  | T7GnaILPd300RIGHT | TGCTTCACTCGCGTTTTTGG | GnaILPd_q_210-422R | CGCTCATGAGTTGATGCGAG |
| *GcorILP4* | T7GnaILPe144LEFT | TACCAGATGCCGGGAAACAC | GnaILPe_q_51-130F | GTCTGATACCACTCGCTCCG |
|  | T7GnaILPe407RIGHT | TACTCGGCGTATTCCTCCCA | GnaILPe_q_51-130R | GCCATGCTTCTTCCCAATCG |
| *GcorILP5* | T7GnaILPc50-256LEFT | CGCCCCAAGACGTCGTAAAT | GnaILPc_q_20_123F | TGCTATGTGTTGTGACTGCG |
|  | T7GnaILPc50-256RIGHT | TCCAGCAGCAGTCATCAACA | GnaILPc_q_20_123R | GTGTAGAGCCCCACGACAAA |
| *GcorInR1* | InR_620F | CCATTTGTCCCGGGGATGAA | InR1_q_88F | TTGTGATTCTCTTCGGCGCA |
|  | InR_1017R | ACCGTTCTGCGACTGGAAAT | InR1_q_197R | ATTCCGGATGTCCACGCTTT |
| *GcorInR2* | InR2comRNAi721F-T7 | ACGTGCACCAACAAATGCC | InR2com_q_382-469F | CACATCGGTGGTGGAGTCAA |
|  | InR2comRNAi1217R-T7 | CCCCTACCTTCGCGTTGTT | InR2com_q_382-469R | AAACTGTAGCTCCCATGGCC |
| *Gcorgapdh* |  |  | gapdh-F | ATTTCCAACGCTTCTTGCAC |
|  |  |  | gapdh-R | CCATCACGCCATAATTTTCC |
| *Gcordsx* |  |  | dsxF-L_370-392_site2MF^*^ | TATAGACCCGCATGTCCTGCAGA |
|  |  |  | dsx_R_MFL_1124_site6^*^ | GCAGAAGTCTAGGAGGATCTCGG |
