# Supplementary material for: A specific type of insulin-like peptide regulates the conditional growth of a beetle weapon
Source: PLoS Biol. 2019 Nov 27;17(11):e3000541. doi: 10.1371/journal.pbio.3000541 (PMC6880982; doi:10.1371/journal.pbio.3000541)
Supplement: S1 Fig — The smaller larvae took longer time for prepupation after isolation from stock culture (i.e., high density and abundant food). Male, Y = −1.175X + 9.13, R2 = 0.50, p < 0.001. Female, Y = −1.950X + 11.7, R2 = 0.60, p < 0.001 (regression analysis). (DOCX) [file pbio.3000541.s006.docx]

**
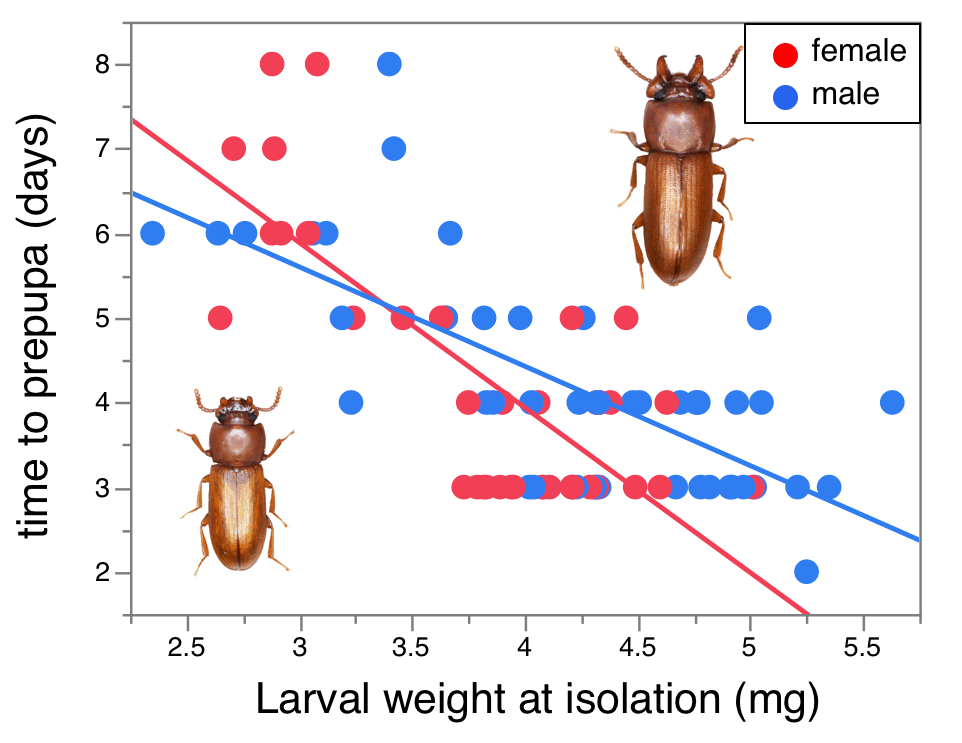
**

**S1 Fig** Developmental schedule depends on larval body size.

The smaller larvae took longer time for prepupation after isolation from stock culture (i.e. high density and abundant food). Male, Y = −1.175X + 9.13, R^2^ = 0.50, *p* < 0.001. Female, Y = −1.950X + 11.7, R^2^ = 0.60, p < 0.001 (regression analysis).
