# Supplementary material for: A specific type of insulin-like peptide regulates the conditional growth of a beetle weapon
Source: PLoS Biol. 2019 Nov 27;17(11):e3000541. doi: 10.1371/journal.pbio.3000541 (PMC6880982; doi:10.1371/journal.pbio.3000541)
Supplement: S2 Fig — Domain-based alignment of (A) insulin-like peptides, (B) IGF-like peptides, and (C) DILP7-like peptides from broad-horned flour beetle (G. cornutus), red flour beetle (T. castaneum), fruit fly (D. melanogaster), yellow fever mosquito (A. aegypti), and honey bee (A. mellifera). Representative ILPs of D. melanogaster (DILP2, -6, -7), A. aegypti (AaegILP3, -5, -6), and A. mellifera (AmILP1, -2) were shown. Highly conserved amino acid residues between all ILPs are shown in red, and highly conserved amino acid residues between orthologous ILPs in G. cornutus and T. castaneum are shown in green. Color bars indicate the predicted domains in the precursor peptides: green, signal peptide; red, B-chain; yellow, C-peptide; blue, A-chain; gray, D-domain. Asterisks on the color bars below the alignment denote Cys residues, and paired triangles denote potential cleavage sites (dibasic amino acids). GcorILP1-4 showed orthologous relationship with TcILP1-4, whereas GcorILP5 has no clear ortholog in T. castaneum. A group of insulin-like peptides (A) shares the most common structural feature of the ILP family, and GcorILP1, -2, -5 are classified into this group. The common feature of this group is a conserved domain organization of their precursors, consisting of a signal peptide, with a B-chain, C-peptide, and A-chain. After cleavage of the signal peptide, the C-peptide is most likely removed to generate a mature heterodimeric peptide consisting of the A- and B-chains like vertebrate insulin. A group of IGF-like peptides (B) is characterized by a relatively shortened or truncated C-peptide like vertebrate IGFs, and GcorILP3 is classified into this group. GcorILP3 has an extended A peptide (D-domain) as seen in TcILP3 and AaegILP6 [30], which are more like the vertebrate IGFs. The third group, DILP7-like peptides (C), is characterized by an unusually conserved sequence shared by several insects, and GcorILP4 is classified into this group. AaegILP, A. aegypti ILP; AmILP, A. mellifera [file pbio.3000541.s007.docx]

**S2 Fig** Predicted prepropeptide structures of insulin-like, IGF-like and DILP7-like peptides in *G. cornutus*.

Domain-based alignment of (A) insulin-like peptides, (B) IGF-like peptides, and (C) DILP7-like peptides from broad-horned flour beetle (*G. cornutus*), red flour beetle (*T. castaneum*), fruit fly (*D. melanogaster*), yellow fever mosquito (*A. aegypti*), and honey bee (*A. mellifera*). Representative ILPs of *D. melanogaster* (DILP2, -6, -7), *A. aegypti* (AaegILP3, -5, -6), and *A. mellifera* (AmILP1, -2) were shown. Highly conserved amino acid residues between all ILPs are shown in red, and highly conserved amino acid residues between orthologous ILPs in *G. cornutus* and *T. castaneum* are shown in green. Color bars indicate the predicted domains in the precursor peptides: green, signal peptide; red, B-chain; yellow, C-peptide; blue, A-chain; gray, D-domain. Asterisks on the color bars below the alignment denote Cys residues, and paired triangles denote potential cleavage sites (dibasic amino acids). GcorILP1-4 showed orthologous relationship with TcILP1-4, whereas GcorILP5 has no clear ortholog in *T. castaneum*. A group of insulin-like peptides (A) shares the most common structural feature of the ILP family, and GcorILP1, -2, -5 are classified into this group. The common feature of this group is a conserved domain organization of their precursors, consisting of a signal peptide, with a B-chain, C-peptide, and A-chain. After cleavage of the signal peptide, the C-peptide is most likely removed to generate a mature heterodimeric peptide consisting of the A- and B-chains like vertebrate insulin. A group of IGF-like peptides (B) is characterized by a relatively shortened or truncated C-peptide like vertebrate IGFs, and GcorILP3 is classified into this group. GcorILP3 has an extended A peptide (D-domain) as seen in TcILP3 and AaegILP6 [30], which are more like the vertebrate IGFs. The third group, DILP7-like peptides (C), is characterized by an unusually conserved sequence shared by several insects, and GcorILP4 is classified into this group. AaegILP, *A. aegypti* ILP; AmILP, *A. mellifera* ILP; DILP, *Drosophila* ILP; GcorILP, *G. cornutus* ILP; IGF, insulin-like growth factor; ILP, insulin-like peptide; TcILP, *T. castaneum* ILP.
