# Supplementary material for: A specific type of insulin-like peptide regulates the conditional growth of a beetle weapon
Source: PLoS Biol. 2019 Nov 27;17(11):e3000541. doi: 10.1371/journal.pbio.3000541 (PMC6880982; doi:10.1371/journal.pbio.3000541)
Supplement: S3 Fig — Four of five GcorILP sequences had close similarities with corresponding TcasILPs, suggesting the orthologous relationships of these four genes. Bootstrap values (%, n = 1,000, maximum likelihood) are shown on the branches. Partial deletion model (90%) with 84 positions were used in final dataset. GcorILP, G. cornutus insulin-like peptide; TcasILP, T. castaneum insulin-like peptide. (DOCX) [file pbio.3000541.s008.docx]

**S3 Fig** Insulin-like peptide phylogeny based on amino acid sequences

Four of five *GcorILP* sequences had close similarities with corresponding *TcasILP*s, suggesting the orthologous relationships of these four genes. Bootstrap values (%, *n* = 1,000, maximum likelihood) are shown on the branches. Partial deletion model (90%) with 84 positions were used in final dataset. *GcorILP*, *G. cornutus* insulin-like peptide; *TcasILP*, *T. castaneum* insulin-like peptide.
