# Supplementary material for: A specific type of insulin-like peptide regulates the conditional growth of a beetle weapon
Source: PLoS Biol. 2019 Nov 27;17(11):e3000541. doi: 10.1371/journal.pbio.3000541 (PMC6880982; doi:10.1371/journal.pbio.3000541)
Supplement: S4 Fig — There are two major two clades in coleopteran InRs (type 1 and type 2 InRs). Twenty amino acid sequences from nine insect species and human were included in the analysis (abbreviations: first letter of genus and first three letters of species, G. cornutus, broad-horned flour beetle; T. castaneum, red flour beetle; Leptinotarsa decemlineata, Colorado potato beetle; O. taurus, dung beetle; Nicrophorus vespilloides, burying beetle; Zootermopsis nevadensis, dampwood termite; A. mellifera, honey bee; D. melanogaster, fruit fly; Nilaparvata lugens, brown planthopper; Homo sapiens, human). Bootstrap values (%, n = 1,000, maximum likelihood) are shown on the branches. Partial deletion model (90%) with 1,086 positions were used in final dataset. IGF, insulin-like growth factor; InR, insulin-like receptor. (DOCX) [file pbio.3000541.s009.docx]

**S4 Fig** Protein phylogeny of insulin-like peptide and IGF receptors.

There are two major two clades in coleopteran InRs (type 1 and type 2 InRs). Twenty amino acid sequences from nine insect species and human were included in the analysis (abbreviations: first letter of genus and first three letters of species, *G. cornutus*, broad-horned flour beetle; *T. castaneum*, red flour beetle; *Leptinotarsa decemlineata*, Colorado potato beetle; *O. taurus*, dung beetle; *Nicrophorus vespilloides*, burying beetle; *Zootermopsis nevadensis*, dampwood termite; *A. mellifera*, honey bee; *D. melanogaster*, fruit fly; *Nilaparvata lugens*, brown planthopper; *Homo sapiens*, human). Bootstrap values (%, *n* = 1,000, maximum likelihood) are shown on the branches. Partial deletion model (90%) with 1,086 positions were used in final dataset. IGF, insulin-like growth factor; InR, insulin-like receptor.
