# Supplementary material for: A specific type of insulin-like peptide regulates the conditional growth of a beetle weapon
Source: PLoS Biol. 2019 Nov 27;17(11):e3000541. doi: 10.1371/journal.pbio.3000541 (PMC6880982; doi:10.1371/journal.pbio.3000541)
Supplement: S5 Fig — GcorILP1,4,5 were predominantly synthetized in brain. GcorILP1 expression was more abundant in small larvae. GcorILP3 was ubiquitously expressed across tissues. Different letters indicate significant differences (GcorILP1,3,5: Steel-Dwass test; GcorILP4: Tukey’s HSD test). Gray: large, blue: small. Day-2 larvae were subjected to analysis. Gene expression levels relative to Gcorgapdh were quantified by qPCR. GcorILP, G. cornutus insulin-like peptide; Gcorgapdh, G. cornutus glyceraldehyde 3-phosphate dehydrogenase; qPCR, quantitative PCR. (DOCX) [file pbio.3000541.s010.docx]

** S5 Fig** Source of *GcorILP1-5* transcripts.

*GcorILP1,4,5* were predominantly synthetized in brain. *GcorILP1* expression was more abundant in small larvae. GcorILP3 was ubiquitously expressed across tissues. Different letters indicate significant differences (*GcorILP1,3,5*: Steel-Dwass test; *GcorILP4*: Tukey’s HSD test). Gray: large, blue: small. Day-2 larvae were subjected to analysis. Gene expression levels relative to *Gcorgapdh* were quantified by qPCR. *GcorILP*, *G. cornutus* insulin-like peptide; *Gcorgapdh*, *G. cornutus* glyceraldehyde 3-phosphate dehydrogenase; qPCR, quantitative PCR.
