# Supplementary material for: A specific type of insulin-like peptide regulates the conditional growth of a beetle weapon
Source: PLoS Biol. 2019 Nov 27;17(11):e3000541. doi: 10.1371/journal.pbio.3000541 (PMC6880982; doi:10.1371/journal.pbio.3000541)
Supplement: S6 Fig — RNAi phenotypes were represented by PC1-PC2 plot. PC1 was a size component positively loaded by all traits, and PC2 was a shape component positively loaded by mandible length, mandible width, and horn length (see S2 Table for factor loadings). Reduced PC2 scores in ILP2RNAi and InR2RNAi. ILP2, insulin-like peptide 2; InR2, insulin-like receptor 2; PC, principal component; RNAi, RNA interference. (DOCX) [file pbio.3000541.s011.docx]

**S6 Fig** Principal component analysis based on sizes of nine body parts.

RNAi phenotypes were represented by PC1-PC2 plot. PC1 was a size component positively loaded by all traits, and PC2 was a shape component positively loaded by mandible length, mandible width and horn length (see Table S2 for factor loadings). Reduced PC2 scores in ILP2^RNAi^ and InR2^RNA^
