# Supplementary material for: A specific type of insulin-like peptide regulates the conditional growth of a beetle weapon
Source: PLoS Biol. 2019 Nov 27;17(11):e3000541. doi: 10.1371/journal.pbio.3000541 (PMC6880982; doi:10.1371/journal.pbio.3000541)
Supplement: S1 Material — GcorILP, G. cornutus insulin-like peptide; GcorInR, G. cornutus insulin-like receptor. (DOCX) [file pbio.3000541.s012.docx]

**S1 Material**  Nucleotide sequences of *GcorILP*s and *GcorInR*s

>Gcor_ILP1

ATGGACAAACGTGTGATATTTTTGGTACTTGTACTCAATGTGATATTCGTGTGGTCTTCC

CCTCACTTGACCCATCTCATGAACAAAAGAGAGTTTTTCTGTGGCAACAAACTCACGGAA

TCGTTAGCTTTGGTGTGTAAAGGCATCTACAACTCTCCAGGCAAAAAATCCTCAATGAAC

GACTTGTTTATCTATGGCGAATATGACGAGCTATTCCCAAATGGCAACGACGACGATAAC

CAATTGGACTTTCCGTTTCTGCAGAAGGAAACGGCGAACTCGTTTGTCCCAGTTAGGTTT

CGAAGGAAAGCTGGAATCGTCGATGAATGTTGCCACAATCCCTGTACTTACAAACACCTC

AGCTTGTACTGCGCATAA

>Gcor_ILP2

ATGGATCTTCAGTGCGTCTTCGTGGTGCTGGCGACGGTGCTGGCGGGCCTCTGCAGCATC

GACGCCGACGAGATGTCGGATTTCAACTCCAAGAAAGTCTATTGCGGCCGCCACCTCTCG

CAGACGCTCTCCGCCGTCTGCAAAGGCAAATACAACACCCTCAACAAGAAGTACGAGACT

AAACCAGAGTCTTTCGGATCTGGGAGCCAAGGCCAGTTTATTACGGACTTAGGATTTCCT

TATCAAACCAAAGCCAGTGCTTCCACTCTCATGACAAACTTTCGGCGTCGGCGGCGTCGC

GGCGTGTTCAACGAGTGCTGTGAGAAACCCTGTTCGCAAGAAGAACTAAAAACGTACTGC

GGCAGCAGAAGACGATAA

>Gcor_ILP3

ATGAACGTGCCAAAAGCATGGATCAAAATTTTGTGTTTGGTGTCCTTGTTCGCTCTCACA

TATGCTAACATCGATTCAAAGGAATTTTTTTGTGGGAAAAAGTTAGTTAGGACGTTGTCG

GAATTATGTTCCATTTACAACAATCCGACCTTTGCTCGCCACCGATTTCGAAGACAGATC

GTGGACGAGTGTTGTCGATCTCAATGCTCCCGTCGCTATCTAGTTCAGTATTATTGCCAA

GAAGCTAACAATTCAGTGGCACATCTCTTGAAAGCCGCCCCCAAAAACGCGAGTGAAGCA

CCTCTCCGTACAAAAGAGCCAGTCCCGGCGCCGCCCAAACCGCGAATGCCCGTAGGTAGC

CCTGAAGATCCTGAAACTAACAATGCTTTTTTGGCAATAGATCTCGCATCAACTCATGAG

CGAAAATTTCATAGGAAACGCAACTGTAAATGCAGAAAGCGCAGGGCAAAGGTGCTATAA

>Gcor_ILP4

ATGTGGTTTCCGGTTTCAACAATAGCGGCTCTTTGCGTTCTTCTTGATGTGTCTGATACC

ACTCGCTCCGATAATGATTTGGAACTTGTCTTTAGAGATAGATCTCAATCCGATTGGGAA

GAAGCATGGCATAAAGAAAAATATACCAGATGCCGGGAAACACTAATCAGACACTTATAC

TGGGCCTGCGAAAAGGACATTTACCGCCTAACCCGAAGAAGCGAACCCAACACTTACACC

AATTACGTACAAAACTATGACTATGAAGAAGATTTCCCATGGTTGGCCCCGAAAAAAGCG

AAAAAACTACTACGTTTCAAACGAGGTCTAAACCGACGATCTGGGTCCTCAATTACTGCA

GAATGTTGTAAATCTTCTGGCTGCACTTGGGAGGAATACGCCGAGTATTGTCCGACTAAT

AAACGATACACGTCGTATGTTTAA

>Gcor_ILP5

ATGAACAAAGTCGTATTATTGCTATGTGTTGTGACTGCGATTTATGCTTCGCCCCAAGAC

GTCGTAAATTATCTAAAAGGCGATTTTAGCCGGCAGTGTTCTTTTTGTCGTGGGGCTCTA

CACGATGCCATGAGAGCAGCGTGTGATGGCTACTACAACACAGTAAACAAGAAGTCGGTT

ATCGATTTGTTTGATGAGGAATTTACGTTTTTTGAGAAGAAATCTGTAGAAAAACTTGTT

GATGACTGCTGCTGGAAGCCCTGTAATTACACTGTGCTCAGGAAGTACTGTTTTTAA

>Gcor_InR1

ATGGCCGCGACCGGCACTGCCTCCACGGCCCAACAGCGCCGCTCCTTCAGCTGGCTCGGC

CCAGGGCCGGTGCTCCACGCGGCTGCTCTTGTGATTCTCTTCGGCGCAGCTTTTGCTAGC

GCAAACCAACTGCAACCTAATTCTGACCTCGCACATCAGAATGGCGGTGAAATTTGCGAA

AGCGTGGACATCCGGAATACATTGGACACGTTTAATCGCCTGAAAGGTTGCCACGTGGTG

GAGGGTTTTGTACAGATTTTACTGTTTGATAATGTAGATGAGCAAGAACTATCCCGCTTA

AGTTTTCCAAACCTAACTGAAATAACAGACTATTTATTATTATACAGAGTGAATGGACTG

AGAAGTATTGGGACTTTGTTCCCAAACTTGAGCGTAATTCGAGGACGAAACACCTTTTAT

ACATATTCTTTCGTTGTTTTCGAAATGTCCACGTTACAAGAAATTGGTTTATATTCTTTG

ACTAACATAACCCATGGATTTATACGAATCGACAAAAATCCTTCATTATGTTTCGTTAAT

TCGATCGATTGGGACTTGATCGCCCATGAGAAAGGAGGAGAACATTTTATAAAAAGCTTA

AAACCAGCGAATGAGTGTCCCATTTGTCCCGGGGATGAAAAAGAAGACGAGTTCAGCAAC

AGTGTTTCCTATACGTCTTGCCCAAAAGCACATCTAAGATCAAACAGCGAGAGTTTCGAA

AGAGATGCTCGCTTATGCTGGAACAGACAACATTGCCAGAGAATTTGCCCGAAAAAATGT

GAACATGCGTGTAATGCCAAAGGAGTGTGCTGTCATGAAAGTTGCTTAGGTGGATGCAGC

TTGGATGACCCCAAACTTTGCACTGTGTGTAAGAACCTCAGCATGGGATTTGGAGCCAAT

AAGCAATGCATGTCGAGTTGTCCACCATTTCAGTATAAGTATTTAGAAAGACGCTGTATA

CTAAGGAACGAATGTAAAGAGATGCCGAAACCGCTTAATTTCCAGTCGCAGAACGGTTCG

GACAAACCGTACAGGATTTTCAACGACTCCTGTCTATTGGAGTGTCCAGCGAACTACACG

AACGATGGCACTTATTGCATCCCATGTAACGGAACTTGCATCAAGAATTGTTCAGGAGTC

AATGTTGACAGCGTAGCGCTAGCAAAACAACTAAAAGGCTGCACTCACATAACCAGTTCT

CTTGAAATTCAGATCCGCGGAGGAAGAAACGTCGTCAACGAGCTCGAAGAAAGCTTGGGA

ATGATCGAGGAAATCGATGGATACCTGAAAGTAGTGAGGTCTTTCCCTTTGGTGTCGCTG

AACTTCTTAAAGAACCTTAAAGTAATCCACGGTAACCAGCTAGAGAGTCAAAAGTACGTT

TTCGTCGTTCTTGACAACCAGAACCTCCAAGAACTGTGGGATTGGGAAAAGAAGGATCTT

AGGATCACCAACGGCCGTTTGTTCTTTCATTTTAACCCAAAACTGTGCTTAAAAGAGATT

AGAAAACTGGAAAAGAAAACGAACATGACCAACGTCACGGAAATAGAAGTGGCCCCGAAT

TCGAACGGTGACAAGATCGCATGTGATTTAAGAGAGTTAAAATTAGAAGGACCTAAGGTT

ATACGTAGTAAAGGAGTTGTCTTGGAGTGGGACCAATTTAAGATGGATGATACTCGAAAA

TTACTCGGTTACATCGTTTTTTATATAGAAGCACCAACGCGGAACGTTACTTTGTACGAT

GGACGAGACGCTTGTGGAGGCGATGGCTGGAGAGTGGATGATGTTACTGTATCGGAAAAC

GATACAAAAGTGACACATCCTTTAATGCGACTAAAGCCATACACTCAGTATGCGTTTTAT

GTAAAGACCTACACCATCGCTACTGAACGGAGAGGTGCCCAGAGCAACATTTCTTACTTC

ACTACTCTTCCTGATACTCCAACTCCGCCGATTTCTCTACAAGTCACGCGGAATTCTAGC

AACTCTTTTCTGATAATGTGGAAGCCGCCAAGAAACCCCAATGGAAATCTCACCCATTAC

ATTGTCAGCGGAAAGATTTACAACACTAATGATAATTCCATTGATTTGGATCATTCTTGC

AAACAGCCGTCGGGTCGCCAGAAACATTCAATGTCTAGTGCCACATCCCCACCAACAGCA

CCATCACTTGCCAGTAATGAAACTTGCCAGTGTCTTGATCCCAAATCGTCAACTTCTAGT

AGCATCAACGAGTATGTCGAGACATCTCGAATTTATTTTGAAGATGCTCTTCACAATGCG

GTTTACATCAAGAAGACCGACACCTCCGGGTCTCGGAAAAAACGTGAGACGCTTTCCTTT

CCCACAGAGGATCAAACTCAAAAAACGAATAGTTATGACATACGTAATGAGACGGATCCA

AAGAGCGGTCGTTGGATTTCTTTTAGCTTTAAAGTTGAGGGAAGAACGGACAAGTACGTC

GGAAATCTCCAACATTTTGCTGCTTACCAAATCAGTGTGCGGGCGTGTAGGGAAAAAAGC

AGCAGTAAGGAAGACAAAACGGAGCCTTGCAGTAATACGAGTTTGAACACGTACAAGACG

CTGAAGAAGCACGATGCTGATGATATCAAGCAGGTTCAAGTTACTAATCAGAGTTTGGAT

ATGGTGTCAATTACGTGGGAAGTGCCTAAAGATCCGAATGGAATAATACTCTGCTATACT

ATTGAGTACAAAAAATTGGATAACGAAAATGCCAAAGCTAATGAAGAGTACGTGAGTCAT

ACCCGATTTTTGAACCAAAGTAGGATCTACACGTTGAAATCTCTTTTACCTGGTAACTAC

AGTTTGAGAGTTCTTGCCACGTCGTCGGCCGACGATGGCGCTTATTCTCCTTATGCGTAC

TTCTACATCGAAGAACAATCTTCTTACACACCGGTAGCGATAACGTTTTCTCTTTTGATT

CCTCTAATTGTTGCTTTATGCGGTGGATTGTGGTGGTATAGGAGAAAAAAAGCAGACAGG

GAAAGCATGAGACTAATTCCTTCAGTAAACCCAGAATATGTCCCGAGCGTTTACGTTCCA

GACGAATGGGAAGTTCCGCGGAAGAAAATTGAGTTAAATAGAGAATTAGGTCAAGGAAGT

TTTGGCATGGTGTATGAAGGTCTTGCCCGCGATGTTCGAGGCAAAGCACAGATTAAATGT

GCAGTGAAAACCGTCAATGAACATGCCACGAATAGAGAACGCCTGGAGTTTTTAAATGAG

GCATCTGTAATGAAAGCTTTTGACACGGCTCATGTAGTTAGATTATTAGGAGTAGTATCT

CAGGGTCAGCCAACTCTTGTAATAATGGAGTTGATGGCGAACGGCGATTTGAAGACATAT

CTGCGTTCACACCGTCCAGATGCAGAAATCTTCGATCCCGCAGCTGCACGACATCCTCCA

ACTCTAAAGCAAATCTTACAAATGGCGATCGAAATAGCAGACGGAATGGCTTATTTATCA

GCAAACAAGTTTGTTCATCGTGACTTGGCAGCTAGGAACTGTATGGTAGCCGAAGATTTA

ACAGTAAAAATCGGTGATTTCGGTATGACGCGCGATATTTACGAAACGGACTATTATAGA

AAAGGGACTAAAGGGCTTCTTCCGGTGCGGTGGATGGCACCGGAAAGCCTAAAAGACGGT

GTGTTTACCATTAATAGTGATGTTTGGAGTTACGGAGTGGTGTTGTGGGAGATGGCAACG

CTTGCATCTCAGCCTTACCAAGGGCTATCAAACGATCAAGTTTTGCGGTATGTGATTGAC

GGGGGTGTGATGGAGCGGCCGGAAAATTGTCCTGATAAACTATACACATTAATGAGATAC

TGTTGGCAACATAAACCATCAGCACGTCCTTCATTCTTAAAGTTATGCTCATTGTTATTA

GAAGATGCCAGCACGAGTTTTGCTCAGGTGTCATTTTATCATAGCGCAGCGGGAATTGAA

GCTCGATCAACGCGTCCAAATCCATCTCCTTCACAGGACGATCCTAGTACTCCTCTTCGG

ATCGCTGATGATCATGATGTCAATTTTTCCCTCAATTCCGATGACTCTAATGATGAGTTT

GAGAATGAGGCTGAAACAGATATGCGATATGCCAGTTATCCAACCGAGAACAAAGAAACC

ACTGCGAATGGTTATGTAAGCGGATGTCCTACCAACGGTGCAGCGACGACTCAGTGCTAG

>Gcor_InR2

ATGCACCGCGTCCTAGTGCTTATCGCGTTGGGGTTGATAATAAACCCCATTGGTGCAAAA

GGTAAAATATGTCAATCAATGGACGTGCGAAACGAAGCTTCGGTCCTGGAAAAGTTAAAA

GGGTGCACAATTATCACGGGCAATTTGACGTTCGTGTTGGTCCACAATGAAAGAGACGAA

AATGTCTTCAAGAAGTATTCGTTTCCGGAACTTACGGAAATCTTGGATTTTTTGATTGTG

TTTAGTGTGGAGAAGTTGACCACGTTGCGGGGAATCTTTCCGAATTTAAGGACCATCAGG

GGAAGGAAGTTGTTTTTGGGATTCGCGCTGATCATCTTTAAAGTGCCGCACTTAGAAGAG

ATTGGTCTGACTAATCTGGTGCACATCGGTGGTGGAGTCAAAGTTGACGGTTGTCCAAAT

CTCTGTTACGTTGACACCATAGACTGGGAGGCCATGGGAGCTACAGTTTTAGACCATCCA

ATGGACCAAGCCGCGATGTGTCAGCAGCAATTCTGCCCCAACGAGTGTGGTGGACATTGC

TGGAATAATTACAGTTGTCAGAAATCAAGGACCAAGGCAAACTATTGTCCTTATTACAAA

GACGGTGAAAACTGCGTTACCGAGTGCCCGAAATCAAAGGTCGTGAGCGAAGAAGCCAAA

CAATGTTGGACGAAAGAAGAATGCAAGATTGCCTTCAACGATACTTGGGCATTCAATGGT

ACGTGCACCAACAAATGCCCGCCGTTGTACTACCGAGACCCGACTGTTGAAGGCGGTTGT

TTTTACGCCGGAAAAGACTACAAGCAGCATTGCACAGGAAATGTGACCATCGACAGTTTG

GAAAGTCTCGAGGAGATGTACGGCTGTACCCACATTAACGGTTATTTAGAGCTTCGTATC

GACGATCCGCAAGTTGAACGGGATTTGGAAAAGTACTTGGGAAACATCGTCGTCATCAGC

GGGTACTTACAAATCGGGCGTTCTAAAGTTATCACGTCACTGCAGTTCTTGAGAAATCTC

ACAGTCATTGAAGGAGAGATCCTCACTAATCACACCGATTCCTCTCTTTTTGTTTACGAA

AATAGAAAACTGAAGACGCTTTGGAATATCACCGATGATTTCAAATTAGAAATACGAAGA

GGGAATTTAACTTTCGTCGACAACCCCCAACTGTGTTTGTCAGAGATTGAAAAATTTCAA

CAACGCGAAGGTAGGGGAAGCACGCCGCTGTCTCAGAATTCCAACGGTGATCAATGGATG

TGTAATATAAAGGACATGGCGGTTACCGAAATCGATAACAATCCCAGTAATATAACGTTA

TCATGGACAGCGATAGAGGGCAATATCCTCGGATACACCGTTTTTTACACATCGAAACAG

GGGAAGACAACCGACGAAAATGATGTTTGCTCCGATGAAAAATGGGAGGGGGTTTTTGTC

CACGACAACGTCGCTGTACTAAAAGATTTGACTCCGTTCACCACCTACACGTACTACATT

AGGACTTACATGAAAAATAATCACAGTAATGAAGGTCAACAAACACCAACTTACGAATTC

ACTACAGCGTCAACAGTACCATCAGAGCCAGTGGATGTAACAGTGGAAGCTTTAAACGCC

ACGAGCATCTTAGTGACATGGAGAGCCCCCCAAACACGTAATGGAGTTTTGATCCATTAT

ATCATCAGTATGTACCACGAAAAAGACTACTTACCGACAATCGAGCAAAGAAATTATTGC

AAAGCACCTCACACTTCAGAAATTCCAATTGTCAATAATGTTTCCAAGCTTGCGATAAAG

GAACCCAATTGCTTATGTTTGAGGGATGAGAGAAGGGAATTTGGACCGCCTAAATCGAAG

TTTCTTGATGATTCCAACTACTACAACGAAAAAGCACTTTCTCAACCAGAATCCGATAAA

ATGACAGCCCGAGATATTGGCACAGTCCGAAACAGGGATACAGTGGTGATTCCAGCAAAC

ACGACGTGGTATATCTTCACCAATTTGCAACCTTTTTCCTTGTACGTTTTTTTGGTGACT

GCTTGCAACATCAAGAGTAAGAAAGAAATGGATGAGGTTCAATGTGGTCGCTCGATAATG

GCTTTTCAACGAACAAAAAAAAGTACCACGGAGGATTTAATCAAACACGTCGTTGTAAAA

ATCGATGGGAAAGATGTTATTTTGAAGTGGTCCAAACCTGAAGATCCGAACTCTCATTTG

GTGTCGTACAATATCGAATACAAGCGAACGGATTCCGAACATTCCAAGCAAAGATTCGAA

TGTTTAACCCACAACGAGTTCCACGGTTTTTACAAGATTCCTCAGCTGAGTTCAGGGAAG

TACGCTGTGAGGGTTCGTGCGATTTCTTATGGTGGTTCTGGGGCTTTCAGTGAGTGGAAG

GAGTTTGAAATCGCCAAAGAAACGAACTCTTCTTTTGTCGTCATCATATCGGTCATTGTT

TCATTGTTGATTGCCTTCATTCTGCTAGCCGCTATGTATTGGTACTACAAAAAGAAGCAC

CTAGACGATAGAAACCACCTGATCACCAGCATAAATCCAGATTATGCCGGTCCGATTTAC

ATGGAAGATGAATGGGAGATCGACCGAAAAGACGTGGATATCCTCGCTGAAATCGGTCAA

GGTTCCTTTGGCATGGTCTACGAGGGCTTAATCAAGTCCCGTCGATACCCATGCGCGATA

AAAACAGTCAACGAGAGCGCCTCTACCTCCAGTCGAATGGAGTTCCTCAACGAAGGCTCC

GTCATGAAGAGCTTTAACGATGCGCAGCACGTAATCAAACTCCTCGGTATCGTGTCCCGA

GGGCAACCTCCCCTAGTAATAATGGAGCTGATGGAACGCGGTGATTTGAAATCGTACCTT

CGCCGTTGCCGCGAGCCGTCCCAAAACCTCACCACCAACGAGCTATATCGCATGGCCGCG

GAAATCGCCGACGGTATGGCTTACCTCTCCGCGAAGAAGTACATCCACCGAGATTTGGCC

GCGCGGAATTGCATGGTCGCCGCTGATCGCACTGTGAAAATCGGCGACTTTGGCATGGCC

AGAGACGTTTATGAGACTGATTATTATAAGAAAGACACTGCTGGGTTGCTTCCGGTGCGG

TGGATGGCACCGGAGAGCCTCGCGGACGGTGTCTTCACCTCCGACTCCGAC
